# Assessment of the Diagnostic Performance and Clinical Impact of AI in Hepatic Steatosis: Systematic Review and Meta-Analysis

**DOI:** 10.2196/78310

**Published:** 2026-01-13

**Authors:** Jiamei Song, Dan Liu, Jitong Li, Haoru Cong, Ruixue Deng, Yihan Lu, Jiayi Sun, Jingzhou Zhang

**Affiliations:** 1 College of Traditional Chinese Medicine, Changchun University of Chinese Medicine, No 1035, Boshuo Road Jingyue National High-Tech Industrial Development Zone Changchun City, Changchun, Jilin, 130117, China, 86 13756864698

**Keywords:** artificial intelligence, AI, diagnostic performance, hepatic steatosis, meta-analysis, clinical impact

## Abstract

**Background:**

The global rise of metabolic associated fatty liver disease reflects the urgent need for accurate, noninvasive diagnostic approaches. The invasive nature of liver biopsy and the limited sensitivity of ultrasound in detecting early steatosis highlight a critical diagnostic gap. Artificial intelligence (AI) has emerged as a transformative tool, enabling the automated detection and grading of hepatic steatosis (HS) from medical imaging data.

**Objective:**

This review aims to quantitatively evaluate the diagnostic performance of AI models for HS, explore sources of interstudy heterogeneity, and provide an appraisal of their clinical applicability, translational potential, and the major barriers impeding widespread implementation.

**Methods:**

PubMed, Cochrane Library, Embase, Web of Science, and IEEE Xplore databases were searched until September 24, 2025. Studies using AI for HS diagnosis, meeting predefined PIRT (Patient Selection, Index Test, Reference Standard, Flow and Timing) framework and providing extractable data were included. Diagnostic performance indicators, including sensitivity, specificity, and the area under the summary receiver operating characteristic curve (AUC), were extracted and quantitatively synthesized. Meta-analyses were conducted using a bivariate random effects model. The methodological quality and risk of bias were evaluated using the QUADAS-2 (Quality Assessment of Diagnostic Accuracy Studies 2) tool. Heterogeneity was assessed through the *I*² statistic, bivariate box plots, 95% PIs, and threshold effect analysis. Clinical applicability was examined using the Fagan nomogram and likelihood ratio tests.

**Results:**

A total of 36 eligible studies were identified, of which 33 (comprising 36 cohorts) were included in the subgroup analyses. Results demonstrated excellent diagnostic accuracy of AI models, with a summary sensitivity of 0.95 (95% CI 0.93-0.96), specificity of 0.93 (95% CI 0.91-0.94), and an AUC of 0.98 (95% CI 0.96-0.99). Clinical applicability analysis (positive likelihood ratio >10; negative likelihood ratio <0.1) supported AI’s strong potential for both confirming and excluding HS. However, substantial heterogeneity was observed across studies (I² >75%). According to QUADAS-2, a high risk of bias, particularly in the Patient Selection domain (44.4%), may have contributed to the overestimation of real-world performance. Subgroup analyses showed that deep learning models significantly outperformed traditional machine learning approaches (AUC: 0.98 vs 0.94). Models using ultrasound or histopathology references, retrospective designs, transfer learning, and public datasets achieved the highest accuracy (AUC 0.98-0.99) but contributed to interstudy heterogeneity.

**Conclusions:**

AI demonstrates remarkable potential for noninvasive screening and assessment of HS, especially in primary care. Nonetheless, clinical translation remains limited by performance variability, retrospective designs, lack of external validation, practical barriers such as data privacy and workflow integration. Future studies should prioritize prospective multicenter trials and standardized external validation to bridge the gap between current evidence and clinical application. The key innovation of this review lies in establishing a unified, modality-agnostic analytical framework that integrates evidence beyond single-modality evaluations.

## Introduction

Metabolic-associated fatty liver disease (MAFLD) has emerged as one of the most prevalent chronic liver diseases worldwide, with its pathophysiology intrinsically linked to metabolic syndrome. Affected individuals frequently exhibit concomitant metabolic abnormalities such as central obesity, type 2 diabetes mellitus, and insulin resistance. The disease spectrum of MAFLD represents a continuum ranging from simple hepatic steatosis (HS) to metabolic dysfunction-associated steatohepatitis (MASH), which may progress to hepatic fibrosis, cirrhosis, or hepatocellular carcinoma (HCC) [1]. Therefore, MAFLD is a significant and growing global public health threat [2].

In 2020, an international consensus panel proposed renaming “non-alcoholic fatty liver disease” to “MAFLD” to better reflect its metabolic foundation [3,4]. To ensure the present study focuses on the diagnostic performance of artificial intelligence (AI) for the core pathological feature of HS and to enhance its clinical generalizability, including to populations with mixed etiologies such as concomitant metabolic disorders and alcohol use, we adopted the broader term “MAFLD.” This terminology aligns more closely with real-world clinical practice and provides a consistent framework for AI model training and validation. Epidemiological data estimate the global prevalence of MAFLD at approximately 38%, with substantial regional variation, the highest burdens observed in Latin America, the Middle East, and North Africa [5]. The disease is also increasingly recognized among pediatric and adolescent populations, particularly in individuals with obesity, where prevalence rates have been reported to range from 7% to 14% or higher [6].

Nevertheless, the reported MAFLD prevalence varies markedly across studies, from 5% to 46% [7], reflecting considerable heterogeneity. First, diagnostic methodologies differ. Although liver biopsy remains the histopathological gold standard, its invasiveness limits clinical use, shifting reliance toward multimodal imaging. Noninvasive modalities such as ultrasound and computed tomography (CT) are widely used due to accessibility and low cost, but they lack precision in quantifying HS. Quantitative imaging techniques, including magnetic resonance imaging–proton density fat fraction (MRI-PDFF) [8], controlled attenuation parameter-based transient elastography, and noninvasive analysis [9], offer superior accuracy but are constrained by cost and limited availability. Clinical prediction models such as the Fatty Liver Index [10], Hepatic Steatosis Index [11], and Liver Fat Equation [12] enable noninvasive diagnosis through integration of anthropometric and biochemical parameters. Nevertheless, they remain vulnerable to measurement variability and lack use for longitudinal monitoring. Second, the sensitivity of existing diagnostic modalities in detecting early-stage steatosis (hepatic fat content <5%) remains suboptimal. Conventional ultrasound, in particular, has a high false-negative rate when hepatic fat content falls below 20% [13], leading to underdiagnosis and misdiagnosis in subclinical populations.

Such diagnostic inaccuracies carry serious clinical implications. Patients erroneously classified as having “simple MASLD” but who also exhibit alcohol use disorder have been shown to experience mortality risks exceeding those of individuals with typical alcoholic liver disease [14]. As MAFLD incidence rises globally, associated cirrhosis and HCC cases are also increasing. Failure to achieve early and accurate diagnosis forfeits the therapeutic window during the reversible steatosis stage, allowing progression to MASH and fibrosis. Notably, MAFLD-related HCC may arise in noncirrhotic livers [15], challenging conventional surveillance strategies that primarily target cirrhotic patients. Moreover, MAFLD is an established independent risk factor for cardiovascular disease [16]. This elevated cardiovascular risk persists throughout the disease course and remains heightened even following liver transplantation [17], underscoring the necessity of lifelong risk management.

Recent advances in AI have revolutionized medical image analysis, and hepatology has been no exception. AI-based approaches have demonstrated strong diagnostic performance across multiple hepatic pathologies. For instance, Meng et al [18] developed a VGGNet-based multistage fibrosis classifier, achieving high accuracy across 3 fibrosis grades. Wang et al [19] introduced the Explainable Diagnosis Recommender intelligent diagnostic system, which uses deep learning (DL) to automatically detect hepatic echinococcosis and cysts from CT scans. Xiao et al [20] proposed a ResNet-101-based multimodal model that classified 6 hepatobiliary diseases using slit-lamp and fundus images, outperforming clinicians of varying experience levels. Calderaro et al [21] used a DL model to reclassify combined hepatocellular-cholangiocarcinoma into pure HCC or intrahepatic cholangiocarcinoma with high sensitivity and specificity, yielding predictions consistent with clinical and molecular profiles. Specifically for HS assessment, Yang et al [22] developed a 2-stage DL model that classified four steatosis grades with an overall accuracy of 76.3% and an area under the summary receiver operating characteristic (SROC) curve (AUC) of 0.88, surpassing traditional clinical indices. Similarly, Wang et al [23] employed DL to quantify hepatic fat content by inferring proton density fat fraction (PDFF) from routine T1-weighted magnetic resonance imaging (MRI) images, surpassing the performance of the conventional 2-point Dixon fat-fraction model.

Despite these promising developments, the application of AI in the diagnosis and grading of MAFLD or HS remains at an early stage [24]. Existing systematic reviews have primarily assessed AI performance within individual imaging modalities. A critical gap remains: a comprehensive evaluation of AI’s overall diagnostic efficacy across diverse imaging platforms and a systematic analysis of the key technical and methodological determinants of performance are still lacking.

Therefore, this study, for the first time, uses a bivariate mixed effects model [25] to systematically assess the overall diagnostic performance of AI in imaging-based detection of HS. The primary objectives are: (1) to quantitatively determine the aggregate diagnostic accuracy of AI models in identifying HS; (2) to comprehensively explore the sources of heterogeneity, with particular emphasis on the influence of factors such as algorithm type, reference standard, imaging modality, study design, and data accessibility; and (3) to evaluate the clinical applicability and translational potential of AI-based diagnostic systems, while identifying major barriers to their broad clinical adoption. Through these aims, the present study seeks to generate robust, high-level evidence that transcends the limitations of individual analytical approaches, thereby providing meaningful guidance for future research and clinical practice.

## Methods

### Research Design and Clinical Questions

This study protocol was registered with the PROSPERO International Prospective Register of Systematic Reviews (Registration: CRD420251046862). The research was conducted as per the PRISMA-DTA (Preferred Reporting Items for Systematic Reviews and Meta-Analyses–Diagnostic Test Accuracy) guidelines (Checklist 1) [26].

### Search Strategy

This systematic search was independently designed and conducted by two researchers as per the PRISMA-DTA (Checklist 1) [26]. PubMed, the Cochrane Library, Embase, Web of Science, and IEEE Xplore were retrieved until September 24, 2025. The search strategy was structured around three core concepts: (1) the disease (“HS,” “non-alcoholic fatty liver disease,” and “MAFLD”); (2) the technology (“AI,” “machine learning [ML],” “DL”); and (3) the diagnostic context (“diagnosis,” “detection”).

Keywords within each conceptual category were combined via the OR operator (eg, AI OR DL OR ML), whereas keywords across different categories were linked using the AND operator (eg, AI AND MAFLD AND diagnosis).

The authors of the identified studies were not contacted. Reference lists of all included studies were manually reviewed to identify any additional eligible publications. No restrictions on language or publication date were applied at the database level to maximize search sensitivity. However, non-English records were excluded during subsequent screening. Gray literature, preprints, and unpublished studies were not systematically searched. This decision was made a priori to focus on peer-reviewed, full-text articles that had undergone editorial review, thereby ensuring baseline methodological quality and the availability of sufficient details for data extraction. The complete, reproducible search strings for all databases are provided in Table S1 in Multimedia Appendix 1.

### Screening Process

Two independent reviewers initially screened all retrieved titles and abstracts. After removing duplicate records, studies were deemed eligible for inclusion if they met the following criteria:

- Study content: the research conformed to the predefined PIRT (Patient Selection, Index Test, Reference Standard, Flow and Timing) framework:
  - Patient Selection (P): patients undergoing abdominal imaging or pathological examination for HS assessment.
  - Index Test (I): AI models based on DL or ML, using input images derived from ultrasound, CT, MRI, or pathology.
  - Reference Standard (R): defined by the original studies, including MRI-PDFF, liver biopsy pathology, or expert-graded ultrasound. These reference standards reflect real-world diagnostic diversity and were recognized as potential sources of heterogeneity.
  - Target Condition (T): Diagnosis and grading of HS according to the thresholds and criteria adopted in the included studies, allowing cross-comparison of AI performance across varying diagnostic definitions.
- Data availability: studies had to provide diagnostic contingency data, true positives (TP), true negatives (TN), false positives (FP), and false negatives (FN), or sufficient information to derive diagnostic performance metrics, such as AUC with 95% CIs, sensitivity, specificity, accuracy, and predictive values.

Exclusion criteria were: (1) language: non-English publications; (2) study type: letters, conference abstracts, reviews, or academic papers lacking original data; (3) study subjects: animal or nonhuman research, bioinformatics-based analyses, and predictive modeling studies focused on indices, risks, or associations rather than diagnosis; and (4) data sufficiency: studies without key contingency data or insufficient information to calculate diagnostic performance metrics.

### Data Extraction

Two researchers independently extracted data based on the following domains: (1) study characteristics: first author, publication year, site of data collection, and duration of the study period; (2) study population: total sample size, and demographic characteristics (mean or median age); (3) methodological parameters: accessibility of clinical sample data, diagnostic reference standard, and validation strategy; (4) algorithmic architecture: type of algorithm, classifier employed, and application of transfer learning (TL); and (5) diagnostic efficacy: raw contingency table data, and aggregated diagnostic performance metrics.

### Diagnostic Performance Evaluation and Quality Assessment

Pooled estimates of sensitivity, specificity, and AUC, together with their 95% CIs, were presented using forest plots. Heterogeneity was quantified using the *I²* statistic. The AUC was designated as the primary indicator for overall diagnostic accuracy, as it integrates performance across all thresholds and remains unaffected by any single cut-off point. A SROC curve was constructed following an assessment of the threshold effect using the Spearman correlation coefficient between the logit of sensitivity and the logit of (1-specificity). Heterogeneity and its implications were further visualized via 95% PIs and bivariate boxplots. Potential small-study effects were evaluated using the Deeks funnel plot asymmetry test. Additional diagnostic indicators, including the diagnostic odds ratio (DOR), positive likelihood ratio (LRP), and negative likelihood ratio (LRN), were calculated. Clinical applicability was further examined using a Fagan nomogram, while the distribution of likelihood ratios across studies was illustrated via scatterplots.

Two investigators assessed the risk of bias via the QUADAS-2 (Quality Assessment of Diagnostic Accuracy Studies 2) checklist (Checklist 2) in Rev-Man in terms of PIRT framework [27]. The QUADAS-2 tool, recommended by the Cochrane collaboration, was used to assess the methodological quality and risk of bias of included diagnostic accuracy studies.

### Quality Assurance and Dispute Resolution

All screening, data extraction, and quality assessment procedures were independently conducted by 2 reviewers. Any discrepancies were first resolved through discussion to reach a consensus. When consensus was not achieved, a third senior investigator adjudicated the disagreement to make the final determination. This multilevel process ensured that all extracted data represented unanimous agreement within the research team.

### Subgroup Analysis

The following independent meta-analyses were conducted:

1. AI type (ML versus DL): DL models were defined as those based on multilayer artificial neural networks, such as convolutional neural networks and recurrent neural networks (RNNs). This category included all studies that explicitly reported using DL or identified architectures such as VGG, ResNet, U-Net, DenseNet, or Inception. ML models referred to traditional algorithms that learn from data without relying primarily on deep neural architectures, including support vector machines (SVM), random forests, decision trees, and logistic regression. To explore the potential influence of different algorithmic approaches on diagnostic performance.
2. Reference standards for steatosis grading (MRI-PDFF, liver histopathology, or ultrasound): to determine whether there was a performance gap between models based on noninvasive imaging and those based on the pathological “gold standard.”
3. Imaging modality (ultrasound, CT, or histopathology): to assess how differences in imaging principles, invasiveness, and the diagnostic information scale (macroscopic versus microscopic) affected model performance.
4. Application of TL: TL was used when a study explicitly reported the use of a model pretrained on a large-scale dataset (eg, ImageNet) as the initial framework for feature extraction or model fine-tuning. To evaluate whether this specific technique could improve model performance in small-sample medical datasets.
5. Study design (single-center versus multicenter): to assess the generalizability of models across different data distributions.
6. Study type (prospective vs retrospective): to explore the temporal relationship between data collection and model development and to evaluate the potential impact of selection bias on performance assessment.
7. Data accessibility: to evaluate the effect of study reproducibility and transparency on research outcomes.

### Data Analysis

Given the substantial heterogeneity observed among included studies with respect to patient populations, imaging devices, and AI algorithms, a bivariate mixed effects model was used to derive more accurate and reliable pooled estimates [25]. To ensure the robustness of the meta-analytic results, quantitative synthesis (eg, subgroup analysis) was performed only when at least 3 independent studies, defined as studies conducted by different authors, using distinct experimental protocols, or involving separate participant cohorts, were available. Multiple effect estimates from the same publication were included when they originated from distinct participant cohorts (eg, multicenter datasets or independent validation sets). When multiple model outputs were reported, only the best-performing model or that validated using an independent dataset was retained. Subgroup analyses were not conducted when fewer than three independent studies were available for a given subgroup. All statistical analyses and visualizations were performed via Stata MP 18 (StataCorp LLC). A 2-tailed *P* value <.05 denoted statistical significance.

## Results

### Included Study Description

**Figure 1.**
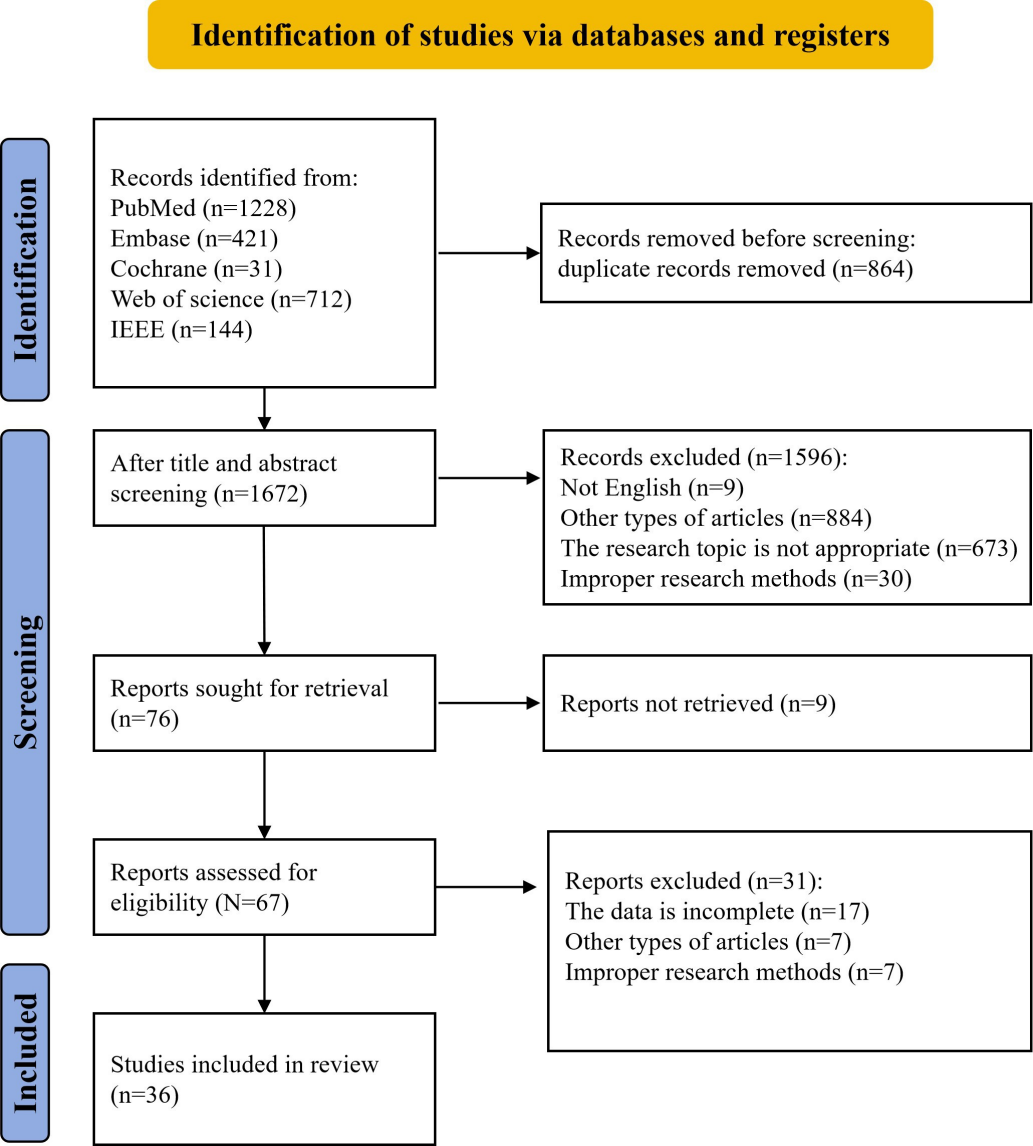
PRISMA (Preferred Reporting Items for Systematic Reviews and Meta-Analyses—Search Extension) flowchart depicting the study selection process for the systematic review of artificial intelligence in diagnosing hepatic steatosis.

As of September 24, 2025, 2536 articles were retrieved. After removing 864 duplicates, the titles and abstracts of the rest were screened as per the predefined eligibility criteria, resulting in the exclusion of 1596 articles. Specifically, 9 were non-English publications, 884 were of other types, 673 involved inappropriate study subjects, and 30 used unsuitable research methods. The full texts of the remaining 76 articles were subsequently reviewed. Seventeen studies were excluded for incomplete data, 7 for being of other types, 7 for inappropriate methodologies, and 9 for being inaccessible. Ultimately, 36 studies were included in the final analysis (Figure 1). The characteristics of the included studies are summarized in Table 1, and the results of the subgroup analyses are presented in Table 2.

**Table 1.** Summary of study characteristics in the systematic review of artificial intelligence (AI)-assisted hepatic steatosis (HS) detection (N=36).

Number	Study	Device	DL^a^ or ML^b^	TL^c^	Source of data	Data range	Sample size	Age, years	Open access data	Research type
1	Yang et al (2022) [28]	MRI^d^	DL	Yes	A hospital in Beijing	Jun‐Jul 2020	50	NR^e^	No	Retrospective study Single-center
2	Acharya et al (2016) [29]	Ultrasound	DL	NR	The University of Malaya Medical Centre, Malaysia	NR	100	NR	No	Retrospective study Single-center
3	Jeon et al (2023) [30]	Ultrasound	DL	NR	Seoul National University Hospital	Jul 2020-Jun 2021	173	mean (SD): 51 (14) range: 19-74	No	Prospective study Single-center
4	Neogi et al (2018) [31]	Ultrasound	DL	NR	Chittaranjan National Cancer Hospital	NR (a span of 6 months)	51	NR	No	Retrospective study Single-center
5	Chen et al (2020) [32]	Ultrasound	DL	NR	Chang Gung Memorial Hospital in Taiwan	2017‐2020	205	mean (SD): 55 (11.6)	No	Retrospective study Single-center
6	Dubois et al (2019) [33]	Ultrasound	DL	NR	Rennes University Hospital	Jun 2017‐Aug 2018	53	median (IQR): 61 (28–72)	No	Prospective study Single-center
7	Shi et al (2019) [34]	Ultrasound	ML	NR	Shanghai Public Health Clinical Center	NR	60	range: 19‐69	No	Retrospective study Single-center
8	Jesper et al (2020) [35]	Ultrasound	ML	NR	Erlangen University Hospital	Oct 2018‐Sep 2019	27	mean (SD): 50 (17)	No	Prospective study Single-center
9	McHenry et al (2020) [36]	MRI	DL	NR	Dallas County	2000-2002; 2007-2009	2139	median: 44	No	Prospective study Single-center
10	Roy et al (2021) [37]	Pathology	DL	NR	Children’s Hospital of Atlanta and Emory University	2014‐2016	36	mean (SD): 14.9 (2.59)	Yes	Retrospective study Multicenter
11	Sun et al (2020) [38]	Pathology	DL	Yes	Washington University School of Medicine Transplant Pathology Service	Apr 2015‐Sep 2016	91	NR	No	Retrospective study Multicenter
12	Chou et al (2021) [39]	Ultrasound	DL	Yes	Taipei Medical University Hospital	2016‐2018	2070	NR	No	Retrospective study Single-center
13	Constantinescu et al (2021) [40]	Ultrasound	DL	Yes	Outpatient clinic of a private healthcare network	NR	60	range: 18‐92	No	Prospective study Single-center
14	Pérez-Sanz et al (2021) [41]	Pathology	DL	NR	University Clinical Hospital Virgen de la Arrixaca-Biomedical Research Institute of Murcia	NR	20	NR	No	Retrospective study Single-center
15	Pickhardt et al (2020) [42]	CT	DL	NR	A single academic medical center	Feb 2010‐Jan 2017	1204	mean (SD): 45.2 (12.4)	No	Retrospective study Single-center
16	Rhyou et al (2021) [43]	Ultrasound	DL	Yes	Samsung Medical Center and Byra Dataset	NR	NR	NR	Yes	Retrospective study Multicenter
17	Destrempes et al (2022) [44]	Ultrasound	ML	NR	Center Hospitalier de l’Universite´ de Montre´al and McGill University Health Center	Oct 2014‐Sep 2018	82	mean (SD): 56 (12) range: 23-78	No	Retrospective study Multicenter
18	Alshagathrh et al (2023) [45]	Ultrasound	DL	Yes	University of Warsaw, Poland	NR	55	mean (SD): 40.1 (9.1)	Yes	Retrospective study Single-center
19	Podder et al (2023) [46]	Pathology	DL	Yes	Open Science Framework	NR	NR	NR	Yes	Retrospective study Single-center
20	Ibrahim et al (2023) [47]	Ultrasound	DL	Yes	Beijing You’an Hospital in Beijing, China, and the National Hepatology and Tropical Medicine Research Institute in Cairo, Egypt	NR	478	mean (SD): 40.97 (10.61)	No	Prospective study Single-center
21	Yao et al (2023) [48]	Ultrasound	DL	Yes	Byra dataset and the Health Service Center in the Chenghua District of Chengdu	2020‐2022	1320	NR	Yes	Retrospective study Multicenter
22	Byra et al (2018) [49]	Ultrasound	DL	Yes	Medical University of Warsaw, Poland	NR	55	mean (SD): 40.1 (9.1)	Yes	Prospective study Multicenter
			23								Torgersen et al (2024) [50]	CT	DL	NR	Philadelphia VA^f^ Medical Center	01 Jan 2010‐30 Dec 2017	120	61.1 (55.3‐64.6)	No	Retrospective study Single-center
24	Wang et al (2023) [51]	Ultrasound	DL	NR	Chang Gung Memorial Hospital, Taiwan	NR	131	Mean (age range) Grade 0: 9 (3-17) Grade 1: 13 (10-17) Grade 2: 11 (4-17) Grade 3: 12 (8-17)	No	Prospective study Single-center
25	Jeon et al (2024) [52]	CT	ML	NR	Institute of Radiation Medicine, Seoul National University Medical Research Center, Seoul National University Hospital, Seoul, Korea	Dec 2018‐Dec 2021	252	mean: 37.3 range: 18-64	No	Retrospective study Single-center
26	Piella et al (2024) [53]	Mobile phones	ML	NR	Vall d’Hebron University Hospital	NR	192	median (IQR): 62 (50.25‐71.75)	No	Retrospective study Single-center
27	Yoo et al (2024) [54]	CT	DL	NR	Radiologic database in our institution	Jan 2017‐Jun 2021	362	mean (SD): 37.3 (11.5) range: 18-65	No	Retrospective study Single-center
28	Zhang et al (2024) [55]	CT	DL	NR	LIDC-IDRI, NSCLC-Lung1, RIDER, VESSEL12, MIDRC-RICORD, COVID-19-Italy, and COVID-19-China	NR	986	NR	Yes	Retrospective study Multicenter
29	Cherchi et al (2021) [56]	Pathology	DL	NR	The University Hospital of Udine	Jan 2018‐May 2019	33	NR	No	Retrospective study Single-center
30	Wu et al (2023) [57]	Ultrasound	DL	NR	Chang Gung Memorial Hospital	NR	276	NR	No	Retrospective study Single-center
31	Drazinos et al (2025) [58]	Ultrasound	DL	NR	The University of Texas MD Anderson Cancer Center	Jan 2018‐Jan 2019	112	mean (SD): 51 (16.13)	No	Retrospective study Single-center
32	Kaffas et al (2025) [59]	Ultrasound	DL	NR	NR	01 Jan 2010‐01 Jan 2022	403	median (IQR): 53 (40‐66)	No	Retrospective study Single-center
33	Kim et al (2025) [60]	CT	DL	NR	Asan Medical Center, University of Ulsan College of Medicine, Seoul, Republic of Korea	2001‐2016	3620	mean (SD): 31.7 (9.4)	No	Retrospective study Single-center
34	Zhang et al (2025) [61]	CT	DL	NR	The First Affiliated Hospital of Zhengzhou University	Jul 2022‐May 2023	840	mean (SD): 49.1 (11.5)	Yes	Retrospective study Single-center
35	Derstine et al (2025) [62]	CT	DL	NR	Michigan Medicine; VA	NR	1740	mean (SD): 43.1 (12.8)	Yes	Retrospective study Single-center
36	Corso et al (2024) [63]	Ultrasound	DL	NR	The University of Texas MD Anderson Cancer Center	NR	186	mean (SD): 51.95 (13.4)	No	Prospective study Multicenter

a DL: deep learning.

b ML: machine learning.

c TL: transfer learning.

d MRI: magnetic resonance imaging.

e NR: No report.

f VA: Veterans Administration.

**Table 2.** Summary of artificial intelligence (AI) performance for diagnosing hepatic steatosis (HS) based on different subgroups (number of studies or cohorts; pooled sensitivity; specificity; area under the curve [AUC]; heterogeneity [*I*², %]; Spearman correlation coefficient [for threshold effect]; and publication bias such as posttest probability positive or negative, positive likelihood ratio [LRP], and negative likelihood ratio [LRN]).

Subgroup and subgroup analysis (studies/datasets)		Pooled sensitivity (95% CI ); *I*² (%)	Pooled specificity (95% CI); *I*² (%)	Summary AUC (95% CI)	Spearman correlation coefficient (*P* value)	Publication bias (*P* value)	Posttest probability positive (%)/ negative (%)	LRP	LRN
AI type									
DL^a^ (29/32)		0.96 (0.94‐0.98); 95.87	0.94 (0.91‐0.95); 97.69	0.98 (0.97‐0.99)	0.21 (*P*=.05)	.46	94/4	>10	<0.1
ML^b^ (4/4)		0.87 (0.78‐0.93); 32.63	0.88 (0.80‐0.93); 63.45	0.94 (0.91‐0.96)	–1 (*P*=.99)	.49	88/13	<10	>0.1
Reference standard									
MRI-PDFF^c^ (7/7)		0.92 (0.86‐0.95); 95.70	0.91 (0.86‐0.94); 98.43	0.97 (0.95‐0.98)	–0.36 (*P*=.13)	.15	91/8	>10	<0.1
Pathology (13/14)		0.97 (0.92‐0.99); 97.86	0.92 (0.86‐0.95); 85.60	0.98 (0.96‐0.99)	0.12 (*P*=.02)	.29	92/3	>10	<0.1
Ultrasound (6/6)		0.98 (0.90‐1.00); 94.32	0.96 (0.94‐0.98); 85.23	0.98 (0.96‐0.99)	1 (*P*=.99)	.21	96/2	>10	<0.1
Imaging modality									
Ultrasound (20/22)		0.96 (0.93‐0.98); 94.86	0.93 (0.90‐0.96); 96.16	0.98 (0.97‐0.99)	0.21 (*P*=.4)	.50	94/4	>10	<0.1
CT^d^ (8/9)		0.93 (0.86‐0.96); 94.43	0.93 (0.87‐0.96); 94.76	0.97 (0.95‐0.98)	0.20 (*P*=.04)	.24	93/7	>10	<0.1
Pathology (4/4)		0.98 (0.91‐1.00); 79.53	0.96 (0.86‐0.99); 0.00	0.99 (0.98‐0.99)	–1 (*P*=.99)	.00	96/2	>10	<0.1
TL^e^									
Used (9/9)		0.99 (0.96‐1.00); 95.36	0.93 (0.88‐0.97); 93.80	0.99 (0.98‐1.00)	0.2 (*P*=.04)	.77	94/1	>10	<0.1
Not used (24/27)		0.93 (0.90‐0.96); 84.22	0.93 (0.90‐0.95); 96.71	0.98 (0.96‐0.99)	0.22 (*P*=.05)	.53	93/7	>10	<0.1
Study design									
Single-center (25/27)		0.94 (0.91‐0.96); 94.44	0.93 (0.91‐0.95); 97.33	0.98 (0.96‐0.99)	0.25 (*P*=.06)	.98	93/6	>10	<0.1
Multicenter (8/9)		0.99 (0.94‐1.00); 95.26	0.92 (0.85‐0.96); 82.33	0.97 (0.96‐0.99)	0.47 (*P*=.22)	.30	92/1	>10	<0.1
Study type									
Retrospective (25/26)		0.95 (0.92‐0.97); 96.58	0.95 (0.92‐0.97); 98.15	0.98 (0.97‐0.99)	0.39 (*P*=.15)	.53	95/5	>10	<0.1
Prospective (8/10)		0.97 (0.92‐0.99); 82.76	0.87 (0.84‐0.89); 53.89	0.90 (0.87‐0.92)	1 (*P*=.99)	.87	88/4	<10	<0.1
Data availability									
Available (9/10)		0.99 (0.96‐1.00); 97.06	0.95 (0.92‐0.97); 73.17	0.99 (0.97‐0.99)	−0.5 (*P*=.25)	.19	96/1	>10	<0.1
Unavailable (24/26)		0.92 (0.89‐0.95); 81.89	0.92 (0.89‐0.94); 96.62	0.97 (0.95‐0.98)	0.09 (*P*=.01)	.30	92/8	>10	<0.1

a DL: deep learning.

b ML: machine learning.

c MRI-PDFF: magnetic resonance imaging–proton density fat fraction.

d CT: computed tomography.

e TL: transfer learning

### Diagnostic Performance and Heterogeneity

**Figure 2.**
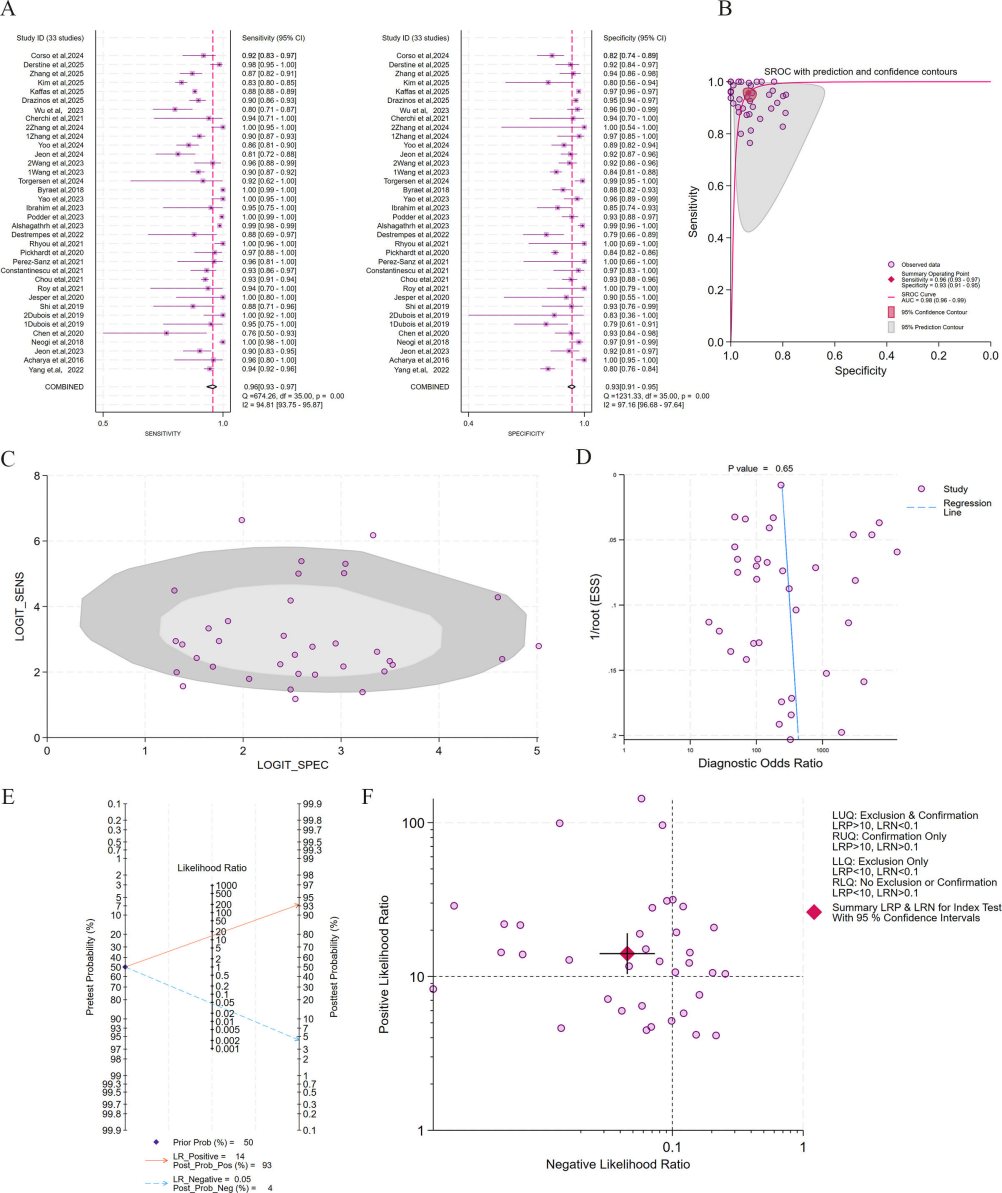
Diagnostic performance of artificial intelligence (AI) models for hepatic steatosis (HS) detection across 33 studies comprising 36 datasets [28-35,37,39-52,54-63]. (A) Forest plots illustrating sensitivity and specificity for the subgroup of AI applications across 33 studies with 36 datasets. (B) Summary receiver operating characteristic (SROC) curve depicting diagnostic performance of AI across 33 studies with 36 datasets, with corresponding 95% CIs. The 95% prediction region reflects the expected range of true sensitivity and specificity in future studies. (C) Bivariate boxplot illustrating the distribution and heterogeneity of AI performance across 33 studies with 36 datasets. (D) The Deeks funnel plot for evaluation of potential publication bias. (E) The Fagan nomogram depicting posttest probabilities. (F) Clinical application plot showing positive likelihood ratio (LRP) and negative likelihood ratio (LRN). LLQ: lower-left quadrant; LUQ: upper-left quadrant; RLQ: lower-right quadrant; RUQ: upper-right quadrant; SROC: summary receiver operating characteristic.

Of the 36 included studies, 33 (comprising 36 datasets) satisfied the criteria for subgroup analysis. The pooled results (Figure 2) demonstrated a summary sensitivity of 0.96 (95% CI 0.93‐0.97), a specificity of 0.93 (95% CI 0.91‐0.95), and an AUC of 0.98 (95% CI 0.96‐0.99), indicating excellent diagnostic discrimination by the AI models. Substantial heterogeneity was observed across studies (*I*²>75%). The Spearman correlation coefficient (0.21, *P*=.05) suggested that threshold effects contributed minimally to overall heterogeneity. The broad 95% PI, however, indicated that differences in diagnostic thresholds were a major source of variability. No significant small-study effects were identified (*P*=.65). In terms of clinical applicability, at a pretest probability of 50%, a positive AI result increased the posttest probability to 93%, whereas a negative result reduced it to 4%. Likelihood ratio scattergram analysis confirmed that the pooled estimates were located within the “confirm and exclude” quadrant (LRP >10 and LRN <0.1), underscoring the strong clinical value of AI for both confirming and excluding HS.

### Risk of Bias Assessment

**Figure 3.**
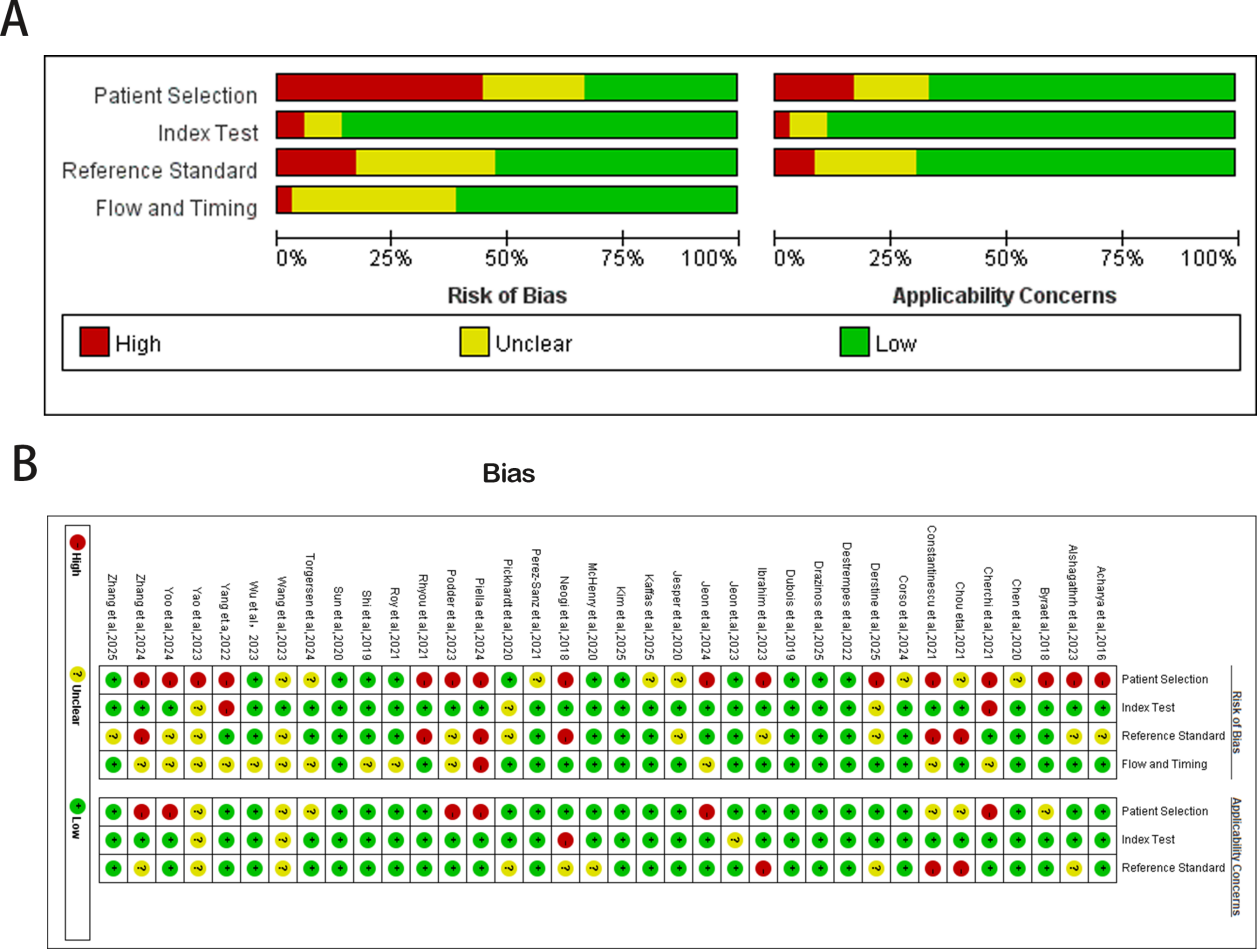
Risk of bias assessment of the 36 included studies on artificial intelligence–based hepatic steatosis diagnosis using the QUADAS-2 (Quality Assessment of Diagnostic Accuracy Studies 2) tool [28-63].

The QUADAS-2 quality assessment (Figure 3) revealed that 44% (16/36) of studies exhibited a high risk of bias in the patient selection domain, primarily due to selection bias, limited representativeness of study populations, and incomplete reporting of key clinical parameters. In the Index test domain, 6% (2/36) of studies were rated as high risk, largely attributable to the absence of image quality control, subjective elements during image processing, and nonstandardized training or validation procedures. In the reference standard domain, 17% (6/36) of studies demonstrated a high risk of bias, most commonly due to deviations from gold-standard reference methods, unclear blinding procedures, or incomplete pathological sampling information. The flow and timing domain exhibited unclear risk in 36% (14/36) of studies, often due to insufficient reporting on patient inclusion pathways and the interval between image acquisition and diagnostic confirmation. These methodological limitations may contribute to an overestimation of AI model performance in real-world clinical practice.

### Subgroup Meta-Analyses

Subgroup analyses were conducted for 7 key variables (Figures S1-S16 in Multimedia Appendix 1), with diagnostic performance interpreted relative to clinical applicability thresholds (LRP >10 for strong rule-in capability; LRN <0.1 for strong rule-out capability).

#### Algorithm Type

**Figure 4.**
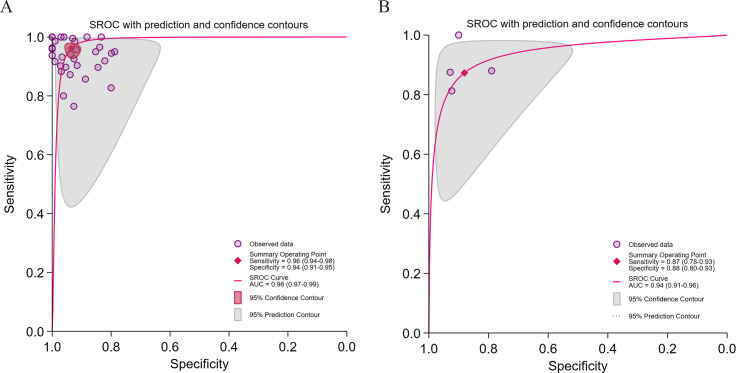
Diagnostic performance stratified by algorithm type for hepatic steatosis (HS) detection. (A) Summary receiver operating characteristic (SROC) curve for deep learning (DL) algorithms in 29 studies [28-33,37,39-43,45-51,54-63] comprising 32 datasets; (B) SROC curve for machine learning (ML) algorithms in 4 studies [34,35,44,52] comprising 4 datasets.

As shown in Figure 4 A-B, the DL models demonstrated significantly higher diagnostic accuracy than ML models (AUC: 0.98 vs 0.94), exhibiting strong rule-in and rule-out performance. However, DL models displayed pronounced heterogeneity (*I*² >95%), likely influenced by threshold effects (Spearman=0.21, *P*=.05), suggesting that these findings should be generalized with caution. ML models showed lower heterogeneity (sensitivity *I*²=32.63%; specificity *I*²=63.45%) but weaker discriminatory power (LRP <10, LRN >0.1).

#### Reference Standard

**Figure 5.**
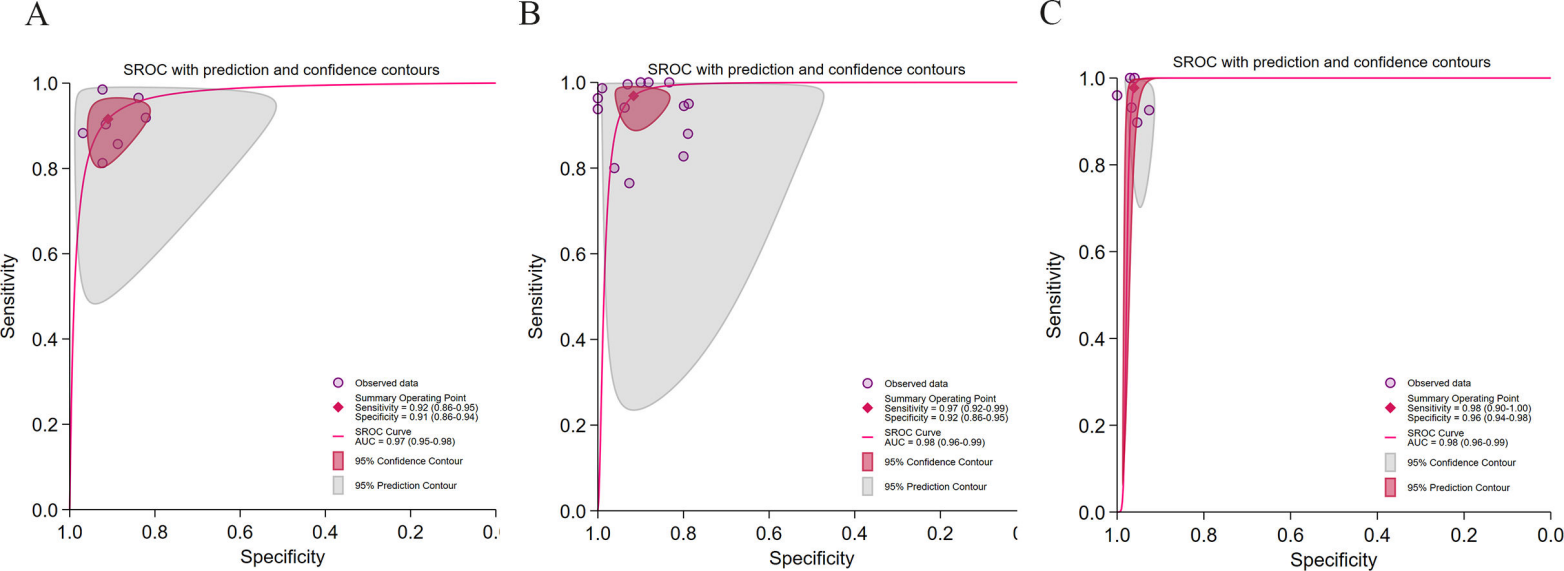
Diagnostic performance stratified by reference standard for hepatic steatosis (HS) detection. (A) Summary receiver operating characteristic (SROC) curve for magnetic resonance imaging–proton density fat fraction (MRI-PDFF) in 7 studies [30,42,52,54,59,62,63] comprising 7 datasets; (B) SROC curve for pathology in 13 studies [28,32,33,35,41,43-46,49,56,57,60] comprising 14 datasets; (C) SROC curve for ultrasound in 6 studies [29,31,39,40,48,58] comprising 6 datasets.

As shown in Figure 5 A-C, studies using ultrasound and histopathology as reference standards achieved comparable AUCs (both were 0.98) with ideal likelihood ratios, although ultrasound-based models exhibited higher pooled sensitivity and specificity (0.98 and 0.96, respectively) than histopathology-based models (0.97 and 0.92). The ultrasound subgroup showed a perfect threshold effect (Spearman=1; *P*=.99), indicating well-defined diagnostic criteria that may be subjectively constrained. The histopathology subgroup exhibited a minimal threshold effect (Spearman=0.12; *P*=.02), suggesting that interstudy variations in sample handling and scoring could significantly influence model performance. The MRI-PDFF subgroup achieved a comparable AUC (0.97) but demonstrated very high heterogeneity (*I*² >95%), limiting result stability.

#### Imaging Modality

**Figure 6.**
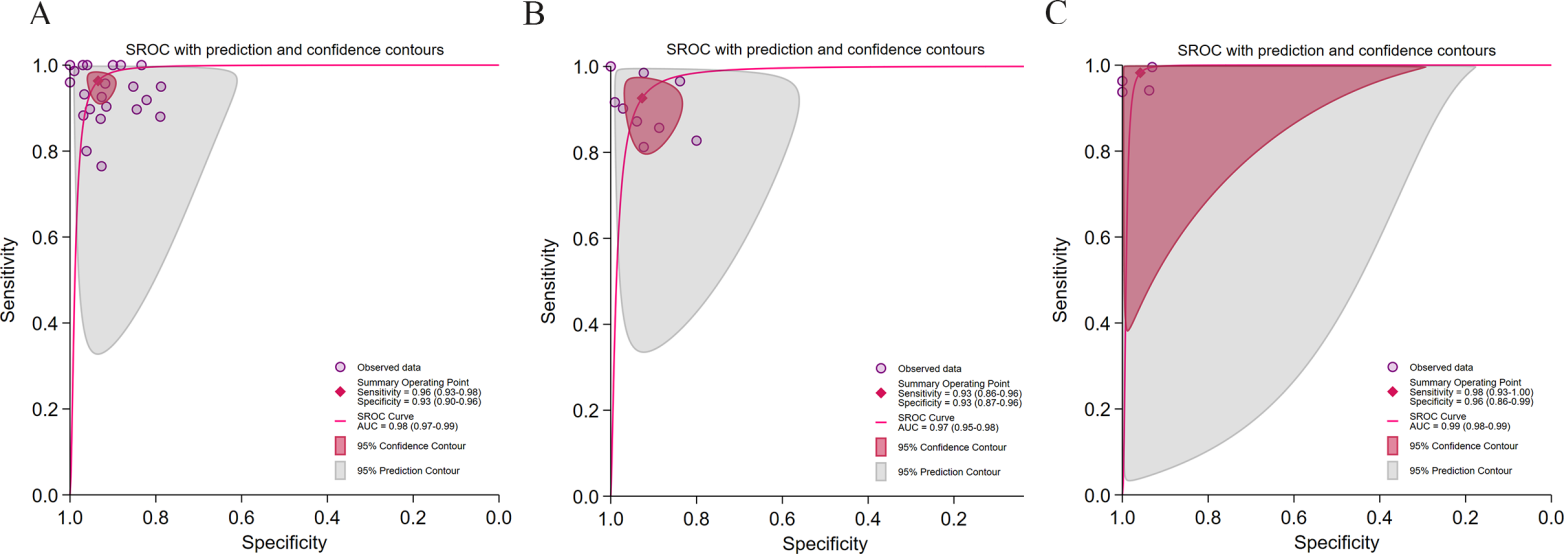
Diagnostic performance stratified by imaging modality for hepatic steatosis (HS) detection. (A) Summary receiver operating characteristic (SROC) curve for ultrasound imaging in 20 studies [29-35,39,40,43-45,47-49,51,57-59,63] comprising 22 datasets; (B) SROC curve for computed tomography (CT) imaging in 8 studies [42,50,52,54,55,60-62] comprising 9 datasets; (C) SROC curve for pathology imaging in 4 studies [37,41,46,56] comprising 4 datasets.

As shown in Figure 6 A-C, histopathology-based models achieved the highest diagnostic performance (AUC=0.99) with no detectable heterogeneity, suggesting robust and consistent results. However, significant publication bias was identified (*P*<.001), implying potential preferential publication of high-performing studies. AI models based on ultrasound and CT achieved comparable accuracy (AUC: 0.98 vs 0.97), though both exhibited marked heterogeneity (*I*² >94%). Only the CT subgroup showed a negligible threshold effect (Spearman=0.20; *P*=.04).

#### Application of TL

**Figure 7.**
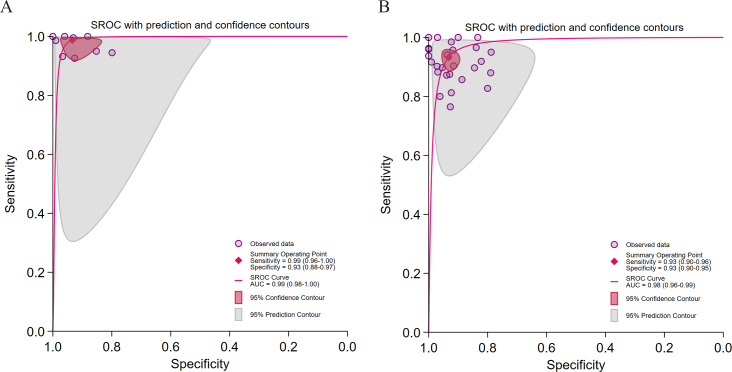
Diagnostic performance of transfer learning (TL) for hepatic steatosis (HS) detection. (A) Summary receiver operating characteristic (SROC) curve for studies employing TL in 9 studies [28,39,40,43,45-49] comprising 9 datasets; (B) SROC curve for studies not employing TL in 24 studies [29-35,37,41,42,44,50-52,54-63] comprising 26 datasets.

As shown in the figure Figure 7A-B models employing TL achieved higher pooled sensitivity (0.99 vs 0.93) and stronger rule-out capability (LRN: 0.01 vs 0.07). No significant publication bias was detected in either subgroup (*P* >.05). Nevertheless, both demonstrated considerable heterogeneity (*I*² >84%) and mild threshold effects (Spearman=0.20 vs 0.22; *P*=.04 vs .05), reflecting the influence of interdomain data discrepancies.

#### Study Design

**Figure 8.**
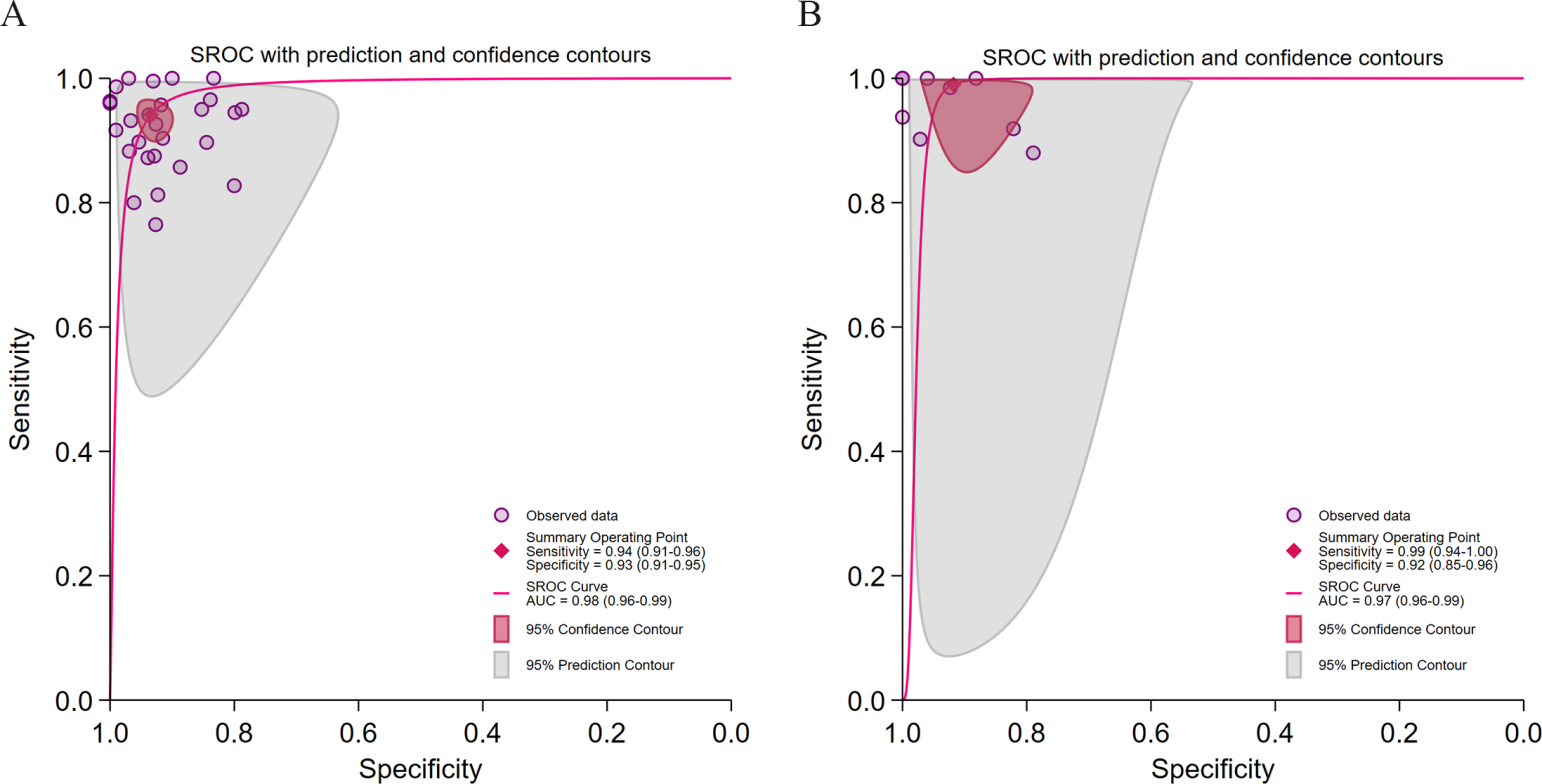
Diagnostic performance of different research designs for hepatic steatosis (HS) detection. (A) Summary receiver operating characteristic (SROC) curve for single-center studies in 25 studies [28-35,39-42,45-47,50-52,54,56-61] with 26 datasets; (B) SROC curve for multicenter studies in 8 studies [37,43,44,48,49,55,62,63] with 9 datasets.

As shown in Figure 8A-B, multicenter studies demonstrated superior sensitivity (0.99 vs 0.94) and lower heterogeneity (*I*²=82.33%), indicating greater generalizability and stronger rule-out potential (LRN: 0.01 vs 0.06). In contrast, single-center studies exhibited marginally higher specificity (0.93 vs 0.92) but very high heterogeneity (*I*² >94%), suggesting limited external validity.

#### Study Type

**Figure 9.**
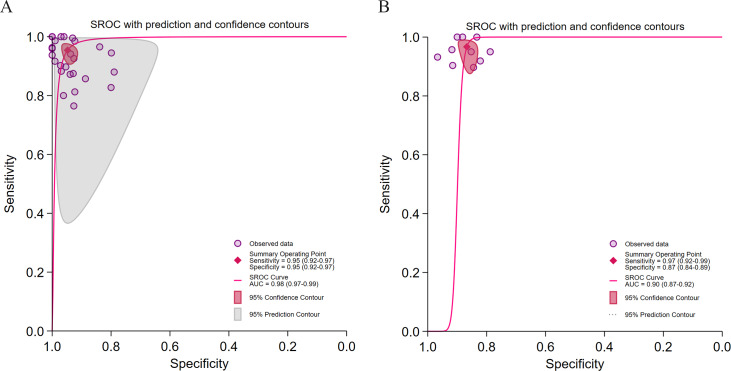
Diagnostic performance of different research types for hepatic steatosis (HS) detection. (A) Summary receiver operating characteristic (SROC) curve for retrospective studies in 25 [28,29,31,32,34,37,41-46,48,50,52,54-62] studies with 26 datasets; (B) SROC curve for prospective studies in 8 studies with 9 datasets. AUC: area under curve.

As shown in Figure 9 A-B, retrospective studies achieved higher overall accuracy (AUC: 0.98 versus 0.90) and stronger rule-in ability (LRP: 9.5 versus 8.8), though with significant heterogeneity (*I*² >96%). Prospective studies, which can better reflect clinical reality, were affected by a perfect threshold effect (Spearman=1) and exhibited weaker rule-in performance (LRP<10).

#### Data Accessibility

**Figure 10.**
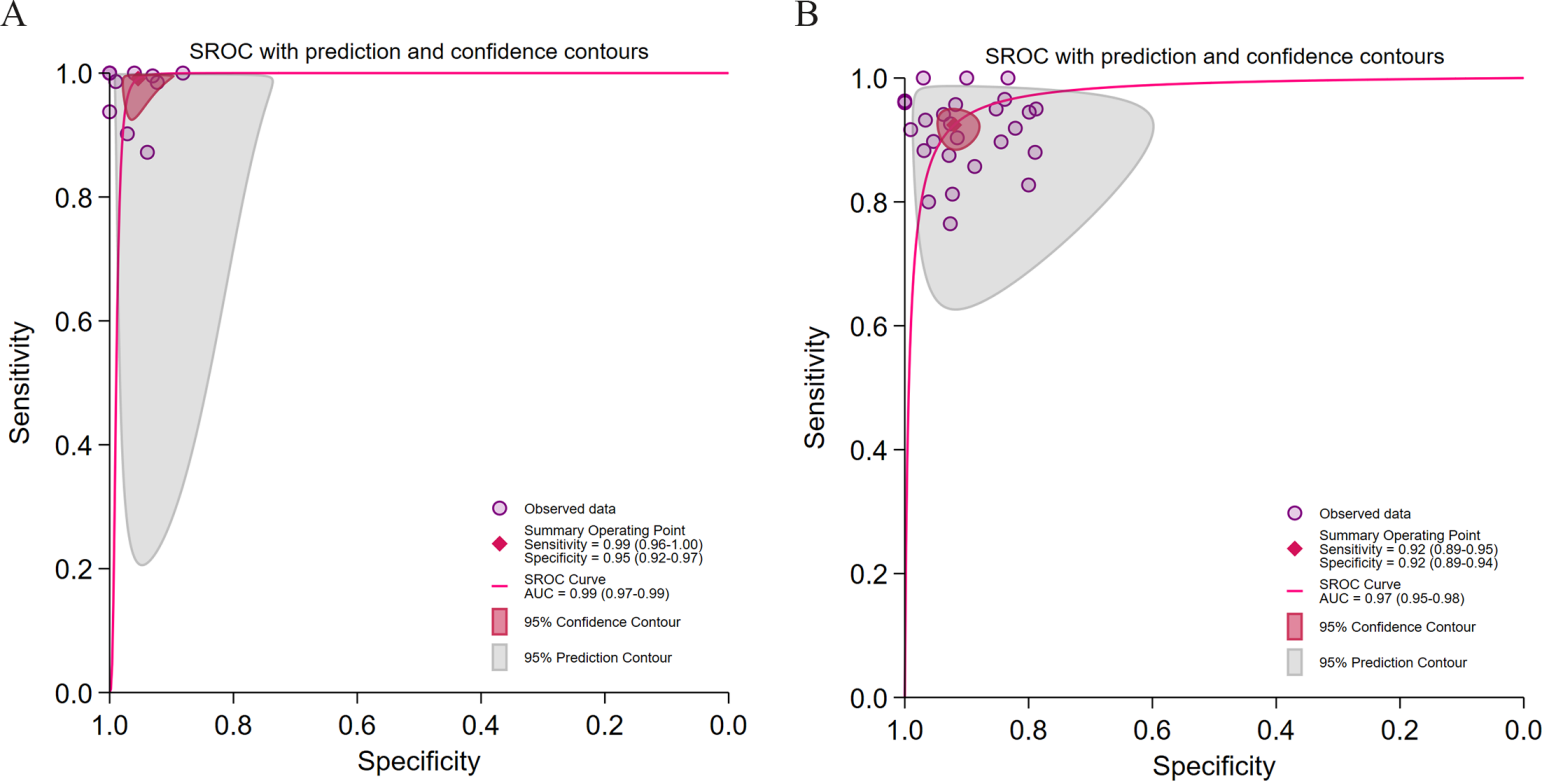
Diagnostic performance of data availability for hepatic steatosis (HS) detection. (A) Summary receiver operating characteristic (SROC) curve for studies with available data in 9 studies [37,43,45,46,48,49,55,61,62] with 10 datasets; (B) SROC curve for studies with unavailable data in 24 studies [28-35,38-42,44,47,50-52,54,56-60,63] with 25 datasets.

As shown in Figure 10A-B studies using publicly available datasets (n=9) achieved superior diagnostic accuracy (AUC=0.99) and stronger clinical applicability (LRP=9.6; LRN=0.01). In contrast, studies using nonpublic data performed comparably (AUC=0.97) but showed a significant threshold effect (Spearman=0.09; *P*=.01), indicating reduced result stability.

Subgroup definitions are detailed in the methods section. Heterogeneity was categorized as follows: *I*²<50% low heterogeneity, 50%‐75% moderate heterogeneity, and >75% high heterogeneity.

## Discussion

### Principal Findings

This meta-analysis of 36 studies demonstrates the superior diagnostic performance of AI in identifying HS, yielding a pooled AUC of 0.98, surpassing that of conventional ultrasound, CT, and MRI, whose pooled AUCs were 0.93, 0.975, and 0.97, respectively [64-66]. These findings underscore AI’s potential to overcome the inherent physical constraints of individual imaging techniques, thereby establishing it as a versatile and adaptive diagnostic approach. This capability carries considerable clinical significance, providing a strong rationale for further meta-analyses dedicated to AI-based diagnostic technologies and informing the development of flexible, context-specific clinical applications. By optimizing the use of the most accessible and cost-effective diagnostic resources, AI could markedly broaden the availability of early HS screening across diverse health care settings.

Although the encouraging performance of AI in diagnosing HS is promising, interpretation of these findings must be tempered by a critical appraisal of the methodological limitations underlying this research. Our analysis revealed substantial heterogeneity (*I*²>75%) and a high overall risk of bias among included studies, particularly within the patient selection domain, where 44% (16/36) were judged to be at high risk. A major limitation lies in the predominance of retrospective, single-center designs (25/36, 69%). Such studies typically develop and validate models within controlled, idealized data environments, meaning that reported metrics may reflect “best-case scenarios” rather than true clinical performance across diverse devices, operators, and patient populations in routine practice. Moreover, independent external validation and multicenter prospective trials remain notably scarce, severely limiting the assessment of these models’ generalizability. Therefore, while existing evidence underscores AI’s considerable potential in HS diagnosis, the current body of research remains insufficient to justify its widespread clinical adoption. Bridging the translational gap from high-performing algorithms to reliable, universally applicable clinical tools thus remains a substantial challenge.

Subgroup analyses provide valuable insights for optimizing AI model design and informing clinical integration. DL-based models demonstrate exceptionally high specificity in diagnosing HS, offering a distinct clinical advantage by reducing unnecessary liver biopsies. However, these models require higher-quality data annotation and greater computational resources. Model performance was also closely associated with the reference standard and imaging modality used. Systems using histopathological images as input achieved the highest diagnostic accuracy. Nevertheless, their clinical applicability is restricted by procedural invasiveness and sampling error [67]. The AI-assisted whole-slide analysis model proposed by Roy et al [37] improves quantitative consistency but faces practical barriers related to cost and patient acceptance.

The choice of imaging modality inherently involves trade-offs between diagnostic accuracy, accessibility, and cost. MRI-PDFF, while providing precise, noninvasive quantification, is affected by confounders such as iron overload, edema, and concurrent pathologies [7], and its high cost limits use in primary care. Ultrasound remains the most accessible and economical option but suffers from operator dependency, reduced sensitivity for mild steatosis, limited penetration in obese individuals [68,69], and suboptimal accuracy in detecting fibrosis [70]. AI integration could mitigate these limitations by standardizing acquisition and interpretation, though at the cost of increased system complexity and computational demand. CT achieves a sensitivity and specificity of 0.93 but is constrained by ionizing radiation exposure and potential interference from iodine-based contrast agents [71]. The dual-energy CT 3D nnU-Net model developed by Yoo et al [54] achieved an AUC of 0.97 for distinguishing steatotic from normal tissue, yet its clinical application is constrained by limited equipment availability.

To optimize model performance and data use, TL has been widely adopted, an especially valuable strategy given the substantial costs associated with medical data annotation. Nonetheless, the effectiveness of TL depends critically on the degree of similarity between the source and target domains; substantial domain discrepancies may lead to “negative transfer,” as illustrated by sensitivity variations of up to 10% in the Inception-v3 model reported by Constantinescu et al [40]. To address this limitation, emerging approaches such as adversarial domain adaptation frameworks have achieved near-human classification accuracy on heterogeneous MRI datasets [72]. Similarly, hybrid pretraining strategies [73] and federated learning techniques have reached up to 99% of the performance attained through centralized training [74]. These approaches enhance model robustness while effectively addressing data privacy and heterogeneity.

Beyond algorithmic optimization, the real-world implementation of AI is profoundly influenced by study design and data governance. Retrospective studies, which constituted the majority (25/36, 69%) of the included reports, demonstrated significantly higher performance than prospective studies (AUC: 0.98 vs 0.94), likely reflecting the high-quality and well-curated imaging data typically available in retrospective cohorts. In contrast, prospective designs more faithfully capture real-world clinical workflows but are inherently subject to operational variability, such as inconsistent imaging protocols and unpredictable patient factors, thereby leading to attenuated performance.

Furthermore, data governance and accessibility are pivotal in determining model generalizability. Multicenter collaborations and data sharing can improve generalizability and reproducibility, though they require standardized imaging protocols, increased logistical coordination, and greater resource investment, posing feasibility challenges in resource-limited settings. Moreover, access to medical data for AI development remains hindered by privacy regulations, institutional policies, and technical interoperability barriers. Privacy-preserving strategies, such as federated learning, offer promising solutions by enabling multi-institutional collaboration without direct data exchange, albeit at the cost of increased computational demands and system complexity. It should also be noted that publicly available datasets may not fully represent the clinical heterogeneity encountered in real-world practice, thereby introducing potential selection bias. These factors, while critical for improving AI performance, also contribute substantially to heterogeneity, underscoring the necessity of comprehensive external validation and context-specific adaptation before large-scale clinical implementation.

### Expanding Role in HS Management

The use of AI extends beyond diagnostic precision to encompass the comprehensive management of HS. Accumulating evidence indicates that AI not only enables accurate quantification of hepatic fat but also integrates radiomic, pathological, and clinical data to facilitate fibrosis staging, predict HCC risk, assess posttransplant survival, and stratify cardiovascular complications. For instance, a VGG16-based ultrasound model outperformed human interpretation in classifying borderline cases [75]. The integration of macrogenomic sequencing with ML has proven effective for the differential diagnosis of HS in obese pediatric populations [76]. Similarly, an ML model based on MRI-derived liver fat quantification markedly improved diagnostic accuracy for liver fibrosis [77]. AI-powered digital pathology platforms reduce the inherent subjectivity of conventional histological assessment [78], while DL-based radiomics facilitates the identification of critical pathological features such as microvascular invasion [79]. A DL algorithm demonstrated 99% accuracy in predicting postliver transplantation survival [80]. In the context of MAFLD-related complications, AI algorithms have been employed to accurately identify affected patients from electronic health records, revealing type 2 diabetes mellitus as a significant predictor of all-cause mortality (hazard ratio: 1.36) [81]. Moreover, a dual model combining tongue imaging with clinical indicators achieved precise prediction of coronary heart disease risk among patients with fatty liver [82]. The foregoing advances signal a diagnostic paradigm shift in HS management from a traditional “liver-centric” approach towards a “patient-centric” model of multi-system risk management, paving the way for early intervention and personalized therapy.

In summary, the advantages of AI in HS diagnosis are threefold as follows:

1. Enhanced early detection: DL models can detect subclinical pathological alterations, including hepatic fat infiltration below 5%, thereby reducing diagnostic subjectivity and improving reproducibility [43,45,83].
2. Standardized quantitative analysis: End-to-end, pixel-level segmentation enables automated calculation of HS, minimizing reliance on manual interpretation and potentially substituting for histopathological assessment in resource-constrained settings**.**
3. Longitudinal predictive modeling: The integration of time-series radiomic and metabolomic features facilitates the construction of individualized models predicting cirrhosis progression and MAFLD onset within 3 years, providing actionable insights for precision treatment planning.

### Challenges and a Phased Implementation Framework

Despite its promising outlook, the widespread clinical adoption of AI in HS management faces multiple challenges. Technically, data heterogeneity, stemming from variations in imaging quality [84], scanner types, and reference standard thresholds, impedes the development of universally robust and generalizable models. Many high-performing algorithms are derived from single-center, retrospective datasets (eg, Yang et al [22], n=50, Beijing) with limited demographic diversity, thereby compromising their external validity and real-world applicability. Moreover, most existing models primarily focus on imaging biomarkers for fat quantification without adequately elucidating the complex pathophysiological interplay among steatosis, metabolic comorbidities, and fibrosis, limiting both clinical interpretability and holistic disease assessment.

From a clinical integration perspective, the transition from algorithmic development to real-world deployment necessitates careful consideration of workflow compatibility, device dependency, and cost-effectiveness. Lightweight AI models hold promise for incorporation into primary care ultrasound systems, facilitating large-scale population screening, whereas more advanced MRI- or CT-based models may be more appropriately implemented in tertiary medical centers. The overarching objective is seamless integration into existing clinical workflows, ensuring that AI serves as an assistive, rather than disruptive, technology that streamlines radiological practice, conserves clinician time, and enhances diagnostic efficiency [85]. Furthermore, issues concerning data privacy [86], algorithmic bias [87], and accountability [88] lack clear regulatory frameworks.

From a global health perspective, the clinical use of AI in HS diagnosis varies according to resource availability. To promote both efficiency and equity in HS diagnosis and management, a phased implementation framework is proposed:

1. Tiered deployment in specific scenarios: in resource-limited settings, lightweight AI systems can be paired with portable ultrasound to enable cost-effective community screening and early detection. Suspected cases may then be referred to higher-level hospitals for precise stratified diagnosis (eg, MRI-PDFF), thereby optimizing resource allocation and minimizing unnecessary biopsies. In high-resource environments, AI-driven automated image processing facilitates accurate fat quantification and disease staging, forming a synergistic diagnostic–therapeutic feedback loop.
2. Establish cross-institutional collaborative data platforms: the adoption of federated learning and related technologies can enhance data diversity while ensuring privacy protection. Such approaches enable robust model development based on heterogeneous real-world data, mitigate model bias and validation gaps, eliminate the need for centralized storage of sensitive information, and provide the foundation for scalable, privacy-preserving deployment.
3. Transition from standalone tools to integrated management platforms: the ultimate objective is to advance AI from a single-function diagnostic aid to a comprehensive, multi-task management system. By synchronously quantifying steatosis, assessing fibrosis, and evaluating inflammatory markers through multimodal data integration. Incorporating these outputs directly into clinical decision-making workflows, AI could evolve from diagnostic assistance to intelligent, holistic disease management.

### Limitations in the Literature

Several limitations warrant cautious interpretation. First, considerable methodological and clinical heterogeneity was observed across the included studies, constraining the reliability of the conclusions. Despite extensive subgroup analyses, variability arising from differences in patient characteristics, imaging equipment, and diagnostic thresholds could not be fully addressed. This residual heterogeneity undermines the robustness of pooled estimates and suggests the influence of unmeasured factors affecting AI performance.

Second, the analysis was limited by methodological shortcomings inherent in the primary studies. Inadequate reporting of key patient characteristics hindered subgroup analyses by disease etiology, particularly distinguishing pure MAFLD from mixed forms, a critical gap given the potential impact of comorbidities on diagnostic accuracy. Furthermore, wide variation in AI architectures and the limited number of comparable models precluded meaningful comparisons across technical approaches, leaving the effect of architectural design on diagnostic performance unclear.

Third, the generalizability and real-world applicability of the findings remain limited. Most studies were retrospective, single-center designs prone to selection bias, with scarce external or temporal validation. Thus, the high-performance metrics reported may represent an idealized best-case scenario rather than outcomes achievable in prospective clinical settings.

Additionally, although our restriction to peer-reviewed full-text publications ensured a baseline level of methodological rigor, the exclusion of relevant preprints and gray literature may have introduced publication bias. Such selective inclusion likely favored studies reporting positive outcomes, potentially leading to overestimated performance measures. Moreover, key practical factors, such as computational burden, workflow integration, and technical expertise, could not be quantitatively evaluated, despite their importance for real-world implementation.

### Conclusions

This meta-analysis highlights the substantial diagnostic potential of AI, particularly DL, in assessing HS. Its key contribution lies in establishing a unified, imaging-modality-independent analytical framework that provides comprehensive evidence beyond the constraints of individual imaging techniques. Nonetheless, these results reflect technical promise rather than confirmed clinical use. The translation from high-performing algorithms to reliable clinical tools remains hindered by performance heterogeneity, retrospective study designs, and insufficient external validation. While the technological foundation of AI in HS is encouraging, clinical maturity has yet to be achieved. Bridging this translational gap will require prospective multicenter studies, standardized reporting protocols, and rigorous external validation. Ultimately, successful clinical adoption will depend on demonstrating not only algorithmic robustness but also tangible improvements in patient outcomes and workflow efficiency across real-world health care settings.

## Supplementary material

10.2196/78310 Multimedia Appendix 1 Retrieval strategy, and Forest plots, bivariate boxplots, Deeks' funnel plots, Fagan's nomogram plots, and clinical application plots for each subgroup analysis.

10.2196/78310 Checklist 1 PRISMA-DTA checklist.

10.2196/78310 Checklist 2 QUADAS-2 checklist.

## Abbreviations

AI: artificial intelligence
AUC: area under curve
DL: deep learning
HCC: hepatocellular carcinoma
HS: hepatic steatosis
LRN: negative likelihood ratio
LRP: positive likelihood ratio
MAFLD: metabolic dysfunction associated fatty liver disease
ML: machine learning
MRI: magnetic resonance imaging
MRI-PDFF: magnetic resonance imaging-proton density fat fraction
PRISMA-DTA: Preferred Reporting Items for Systematic Reviews and Meta-Analyses–Diagnostic Test Accuracy
PRISMA-S: Preferred Reporting Items for Systematic Reviews and Meta-Analyses–Search Extension
SROC: summary receiver operating characteristic
TL: transfer learning
