# Supplementary material for: Assessment of the Diagnostic Performance and Clinical Impact of AI in Hepatic Steatosis: Systematic Review and Meta-Analysis
Source: J Med Internet Res. 2026 Jan 13;28:e78310. doi: 10.2196/78310 (PMC12798848; doi:10.2196/78310)
Supplement: Multimedia Appendix 1 [file jmir-v28-e78310-s001.docx]

**Supplementary Table S1** Retrieval strategy

| Database | Platform / Provider | Date of Search | Search number | Retrieval strategy | Limits / Filters Applied at Search | Results |
| --- | --- | --- | --- | --- | --- | --- |
| PubMed | NCBI | 2025-09-24 | #1 | ((((((Non-alcoholic Fatty Liver Disease) OR (metabolic associated fatty liver disease)) OR (Fatty Liver)) OR (Liver Diseases)) OR (Steatohepatitis)) OR (MAFLD)) OR (NAFLD) | None | 1228 |
|  |  |  | #2 | ((((((machine learning) OR (Deep learning)) OR (artificial intelligence)) OR (artificial neural network)) OR (External validation)) OR (Convolutional Neural Network)) OR (CNN) |  |  |
|  |  |  | #3 | (Hepatic Steatosis) OR (fatty degeneration) |  |  |
|  |  |  | #1 AND #2 AND #3 | |  |  |
| Cochrane library | Wiley | 2025-09-24 | #1 | (Non-alcoholic Fatty Liver Disease):ti,ab,kw OR (metabolic associated fatty liver disease):ti,ab,kw OR (Fatty Liver):ti,ab,kw OR (Liver Diseases):ti,ab,kw OR (Steatohepatitis):ti,ab,kw | None | 31 |
|  |  |  | #2 | (machine learning):ti,ab,kw OR (Deep learning):ti,ab,kw OR (Convolutional Neural Network):ti,ab,kw OR (artificial intelligence):ti,ab,kw OR (External validation):ti,ab,kw |  |  |
|  |  |  | #3 | (Hepatic Steatosis):ti,ab,kw OR (fatty degeneration):ti,ab,kw OR (Steatosis of Liver):ti,ab,kw OR (Visceral Steatosis):ti,ab,kw OR (Liver Steatosis):ti,ab,kw |  |  |
|  |  |  | #1 AND #2 AND #3 | |  |  |
| Embase | Elsevier | 2025-09-24 | #1 | 'hepatic steatosis':ti,ab,kw OR 'fatty degeneration':ti,ab,kw OR 'steatosis of liver':ti,ab,kw OR 'visceral steatosis':ti,ab,kw OR 'liver steatosis':ti,ab,kw | None | 421 |
|  |  |  | #2 | 'machine learning':ti,ab,kw OR 'deep learning':ti,ab,kw OR 'artificial intelligence':ti,ab,kw OR 'artificial neural network':ti,ab,kw OR 'external validation':ti,ab,kw OR 'convolutional neural network':ti,ab,kw OR cnn:ti,ab,kw |  |  |
|  |  |  | #3 | 'non-alcoholic fatty liver disease':ti,ab,kw OR 'metabolic associated fatty liver disease':ti,ab,kw OR 'fatty liver':ti,ab,kw OR 'liver diseases':ti,ab,kw OR steatohepatitis:ti,ab,kw OR nafld:ti,ab,kw OR mafld:ti,ab,kw |  |  |
|  |  |  | #1 AND #2 AND #3 | |  |  |
| Web of Science | Clarivate | 2025-09-24 | #1 | ((((((TS=(machine learning)) OR TS=(Deep learning)) OR TS=(artificial intelligence)) OR TS=(artificial neural network)) OR TS=(External validation)) OR TS=(Convolutional Neural Network)) OR TS=(CNN) and Preprint Citation Index (Exclude – Database) | Source: Preprint Citation Index was excluded. | 712 |
|  |  |  | #2 | ((((TS=(Hepatic Steatosis)) OR TS=(fatty degeneration)) OR TS=(Steatosis of Liver)) OR TS=(Visceral Steatosis)) OR TS=(Liver Steatosis) and Preprint Citation Index (Exclude – Database) |  |  |
|  |  |  | #3 | ((((((TS=(Non-alcoholic Fatty Liver Disease)) OR TS=(metabolic associated fatty liver disease)) OR TS=(Fatty Liver)) OR TS=(Liver Diseases)) OR TS=(Steatohepatitis)) OR TS=(NAFLD)) OR TS=(MAFLD) and Preprint Citation Index (Exclude – Database) |  |  |
|  |  |  | #1 AND #2 AND #3 and Preprint Citation Index (Exclude – Database) | |  |  |
| IEEE Xplore | IEEE | 2025-09-24 | #1 | ((Non-alcoholic Fatty Liver Disease) OR (metabolic associated fatty liver disease) OR (Fatty Liver) OR (Liver Diseases) OR (Steatohepatitis) OR (NAFLD) OR (MAFLD)) AND ((Hepatic Steatosis) OR (fatty degeneration) OR (Steatosis of Liver) OR (Visceral Steatosis) OR (Liver Steatosis)) | None | 144 |
| Other supplementary searches | No additional sources (e.g., grey literature, reference list checking) were searched beyond the electronic databases listed above. | | | | | |

**
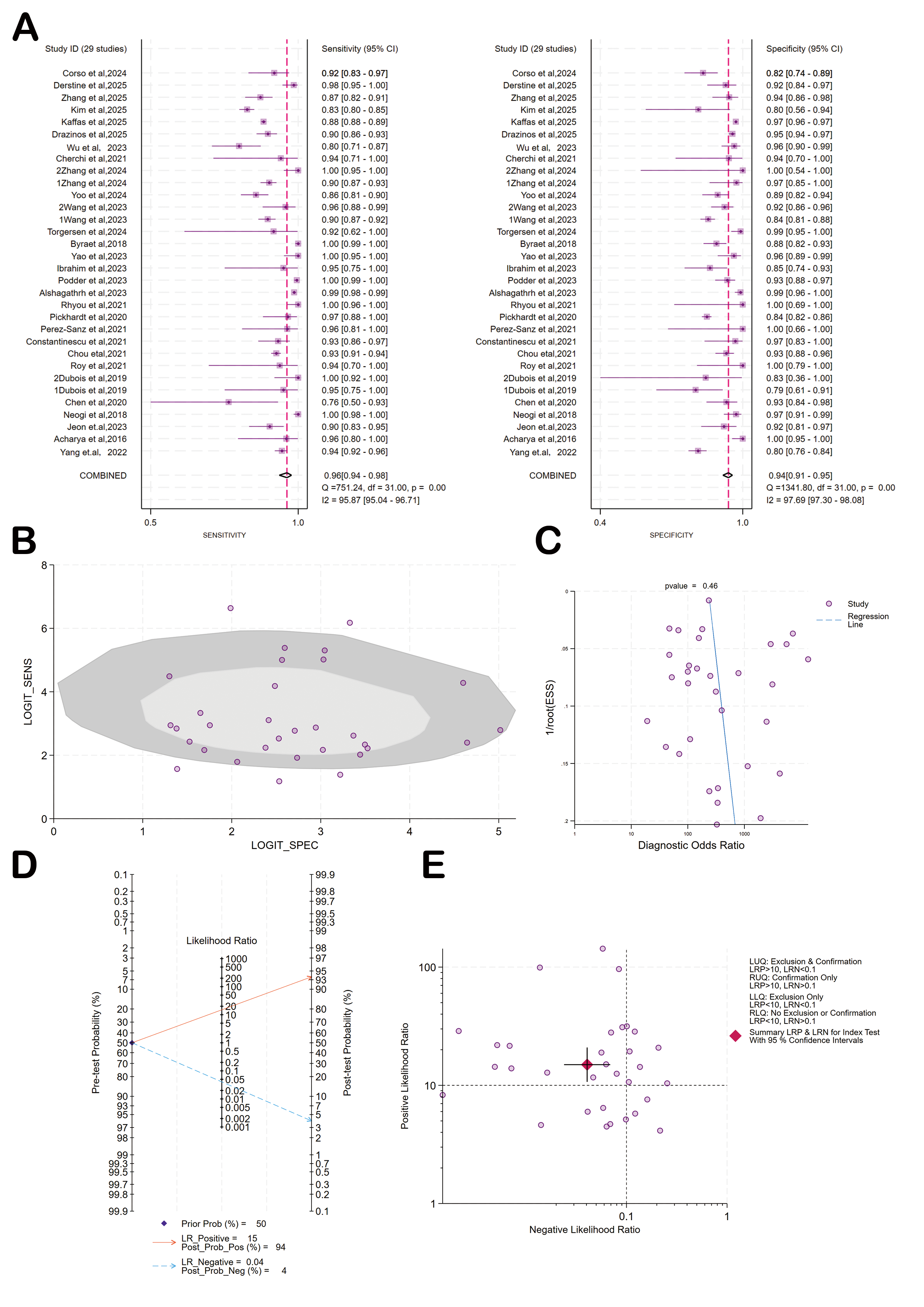
Diagnostic performance summary of deep learning (DL) models: forest plots, bivariate boxplot, Deeks' funnel plot, Fagan's nomogram, and clinical application plot.**

A) Forest plots of sensitivity and specificity for the DL subgroup (29 studies, 32 datasets); B) bivariate boxplot illustrating distribution and heterogeneity; C) Deeks’ funnel plot assessing potential publication bias; D) Fagan’s nomogram depicting post-test probability; E) clinical application plot of LRP and LRN.

**
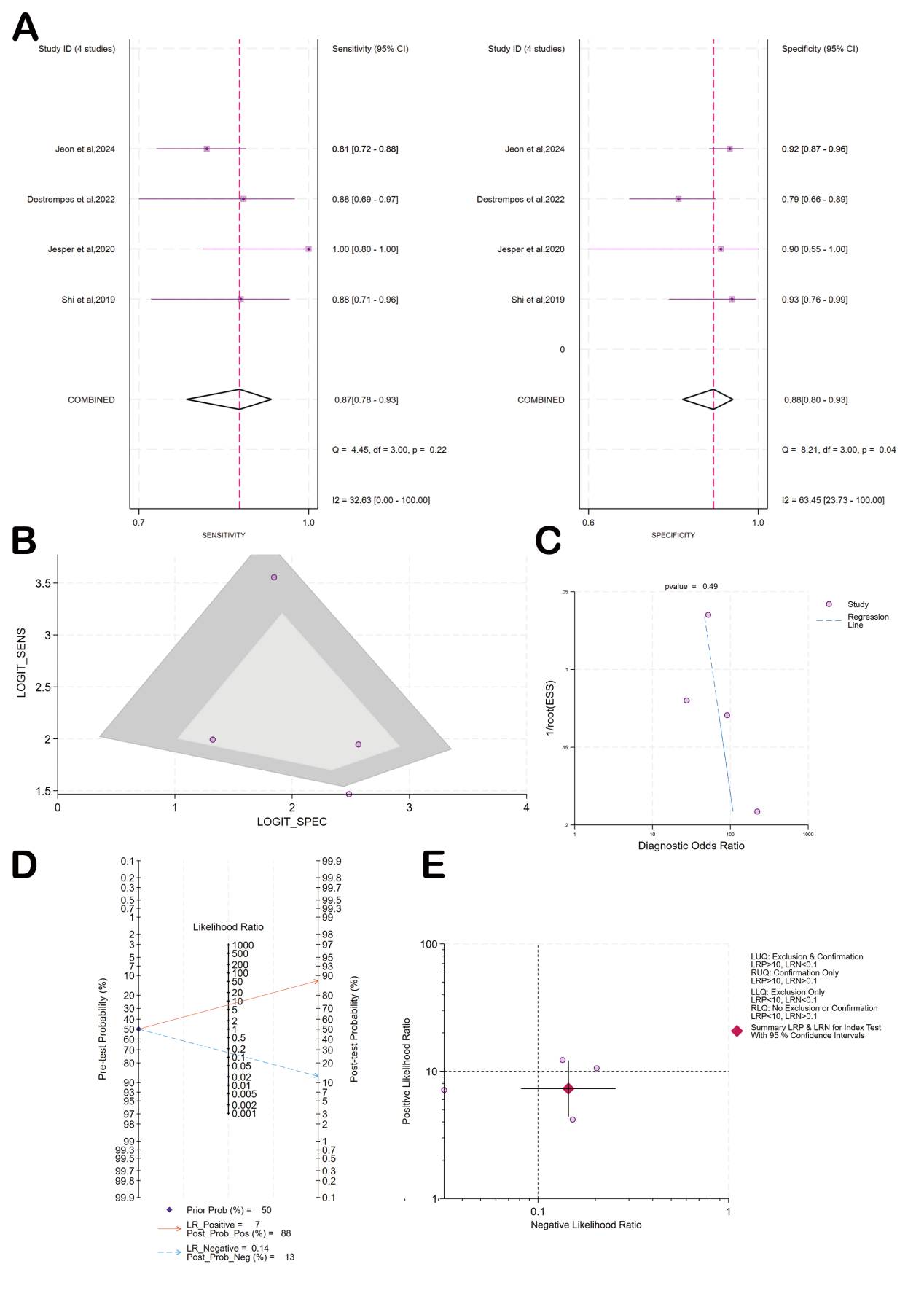
Supplementary Figure S2. Diagnostic performance summary of machine learning (ML) models: forest plots, bivariate boxplot, Deeks' funnel plot, Fagan's nomogram, and clinical application plot.**

A) Forest plots of sensitivity and specificity for the ML subgroup (4 studies, 4 datasets). B) Bivariate boxplot illustrating distribution and heterogeneity. C) Deeks’ funnel plot assessing potential publication bias. D) Fagan’s nomogram depicting post-test probability. E) Clinical application plot of LRP and LRN.

**
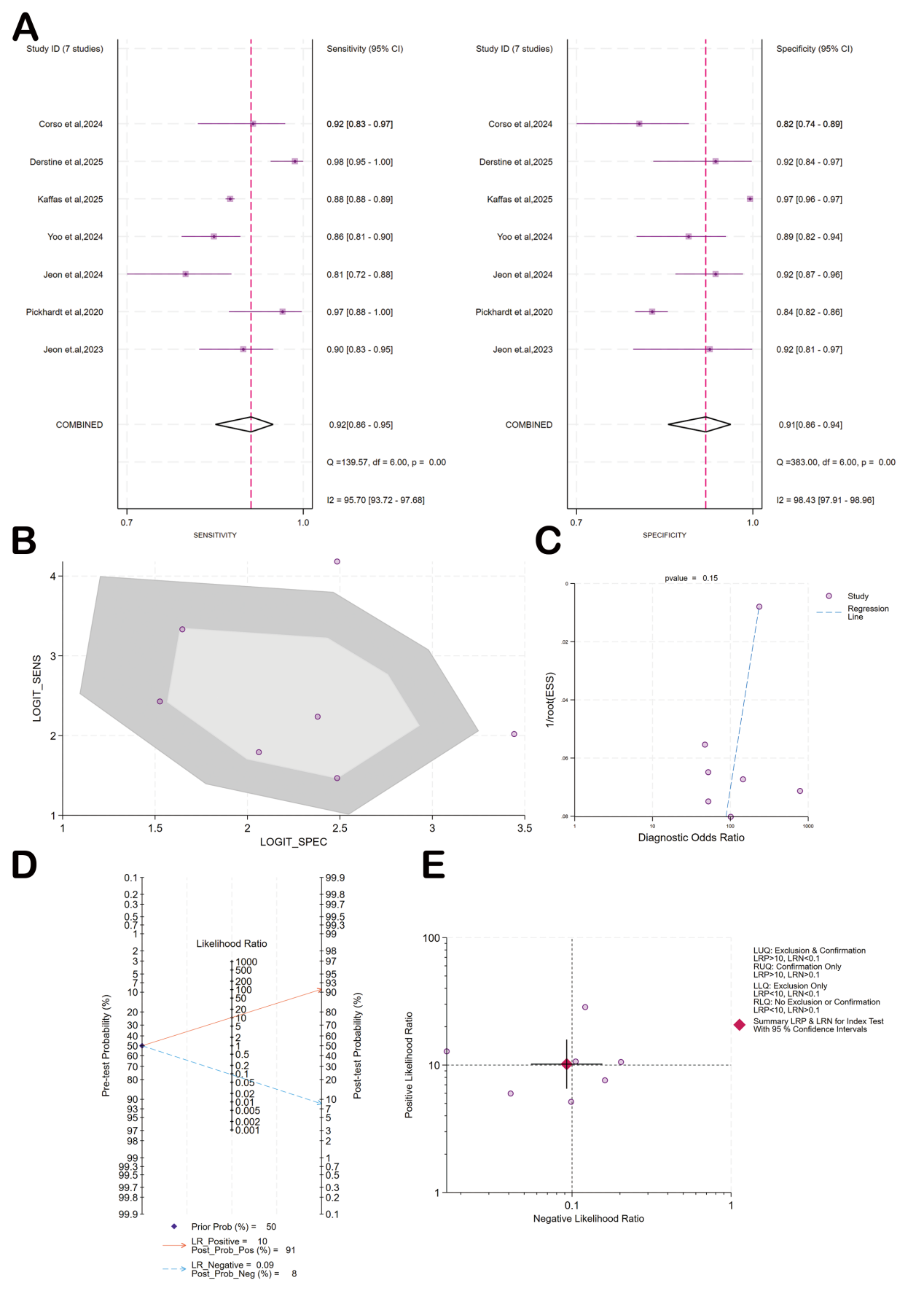
Supplementary Figure S3. Diagnostic performance summary for studies using MRI-PDFF as reference standard: forest plots, bivariate boxplot, Deeks' funnel plot, Fagan's nomogram, and clinical application plot.**

A) Forest plots of sensitivity and specificity for the MRI-PDFF subgroup (7 studies, 7 datasets). B) Bivariate boxplot illustrating distribution and heterogeneity. C) Deeks’ funnel plot assessing potential publication bias. D) Fagan’s nomogram depicting post-test probability. E) Clinical application plot of LRP and LRN.

**
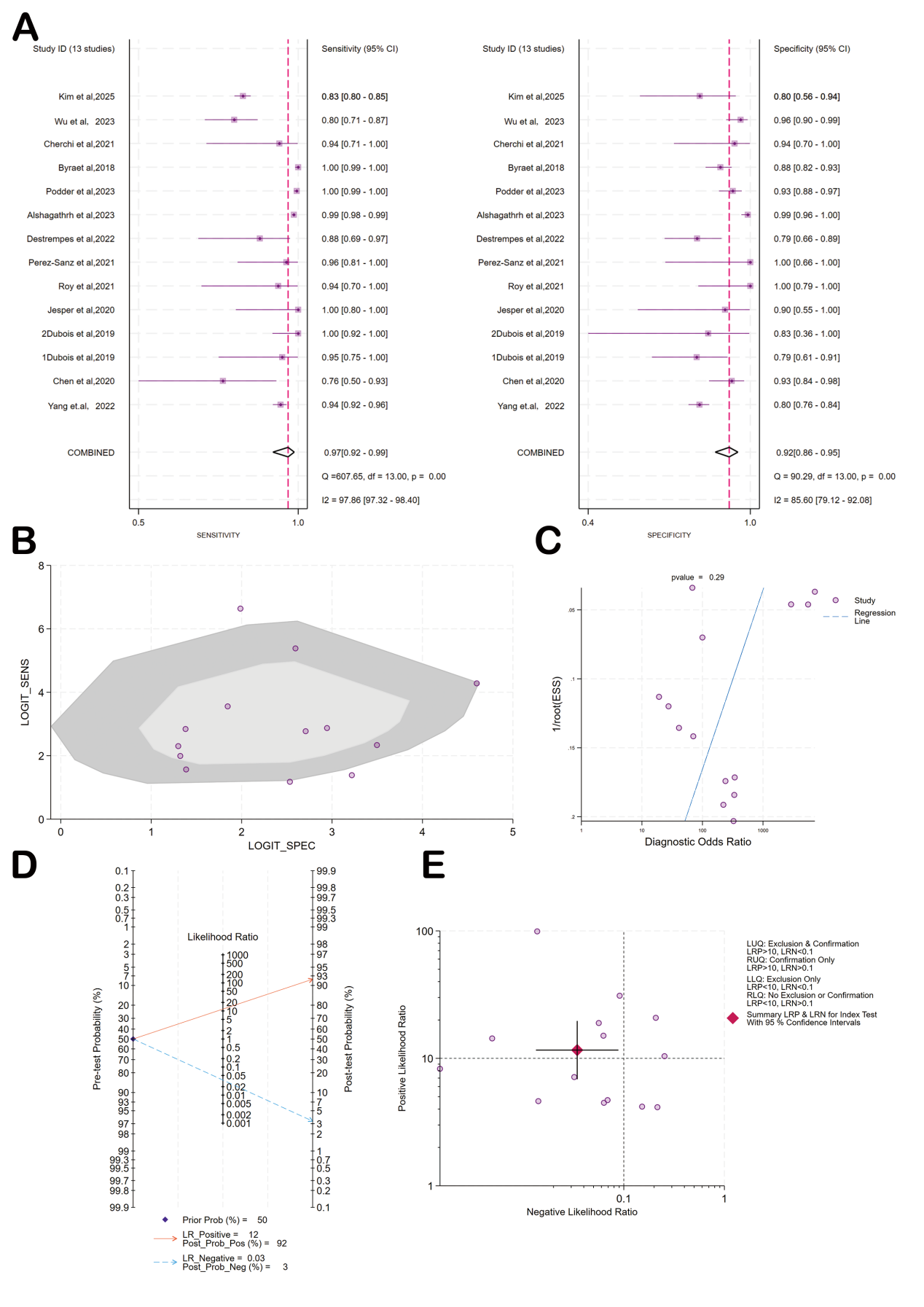
Supplementary Figure S4. Diagnostic performance summary for studies using pathology as reference standard: forest plots, bivariate boxplot, Deeks' funnel plot, Fagan's nomogram, and clinical application plot.**

A) Forest plots of sensitivity and specificity for the pathology subgroup (13 studies, 14 datasets). B) Bivariate boxplot illustrating distribution and heterogeneity. C) Deeks’ funnel plot assessing potential publication bias. D) Fagan’s nomogram depicting post-test probability. E) Clinical application plot of LRP and LRN.

**
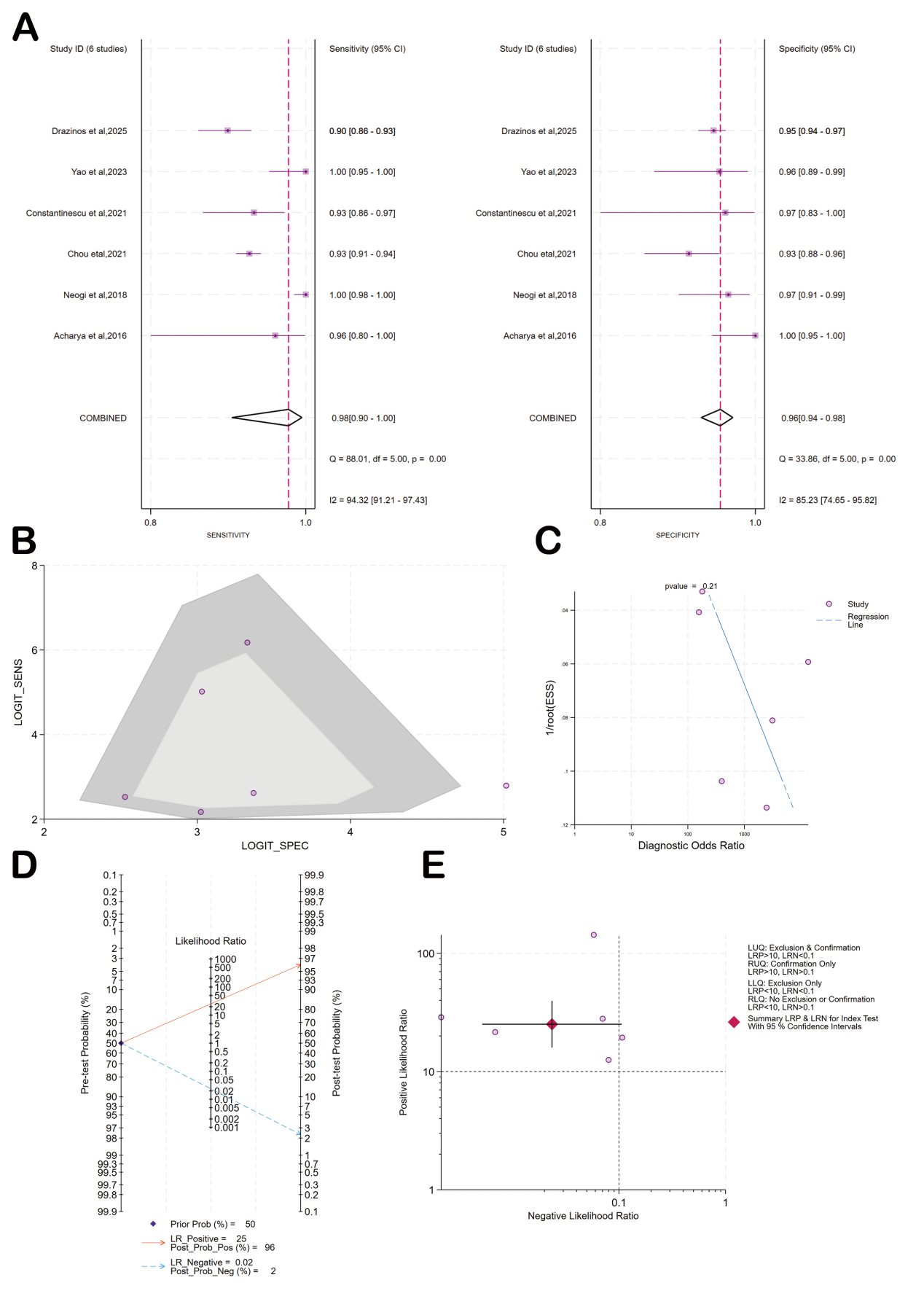
Supplementary Figure S5. Diagnostic performance summary for studies using ultrasound as reference standard: forest plots, bivariate boxplot, Deeks' funnel plot, Fagan's nomogram, and clinical application plot.**

A) Forest plots of sensitivity and specificity for the US subgroup (6 studies, 6 datasets). B) Bivariate boxplot illustrating distribution and heterogeneity. C) Deeks’ funnel plot assessing potential publication bias. D) Fagan’s nomogram depicting post-test probability. E) Clinical application plot of LRP and LRN.

**
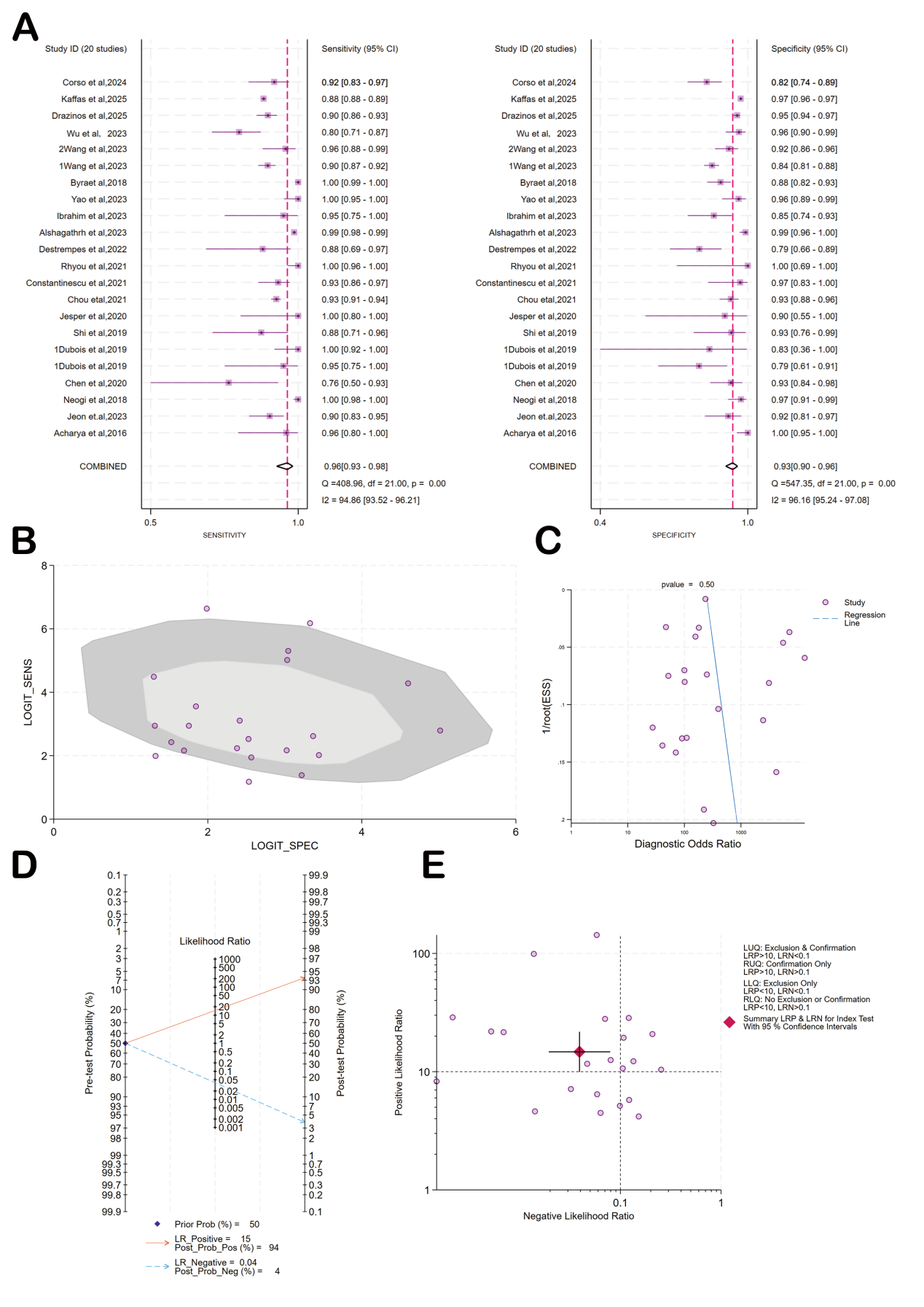
Supplementary Figure S6. Diagnostic performance summary for studies based on ultrasound imaging: forest plots, bivariate boxplot, Deeks' funnel plot, Fagan's nomogram, and clinical application plot.**

A) Forest plots of sensitivity and specificity for US imaging (20 studies, 22 datasets). B) Bivariate boxplot illustrating distribution and heterogeneity. C) Deeks’ funnel plot assessing potential publication bias. D) Fagan’s nomogram depicting post-test probability. E) Clinical application plot of LRP and LRN.

**
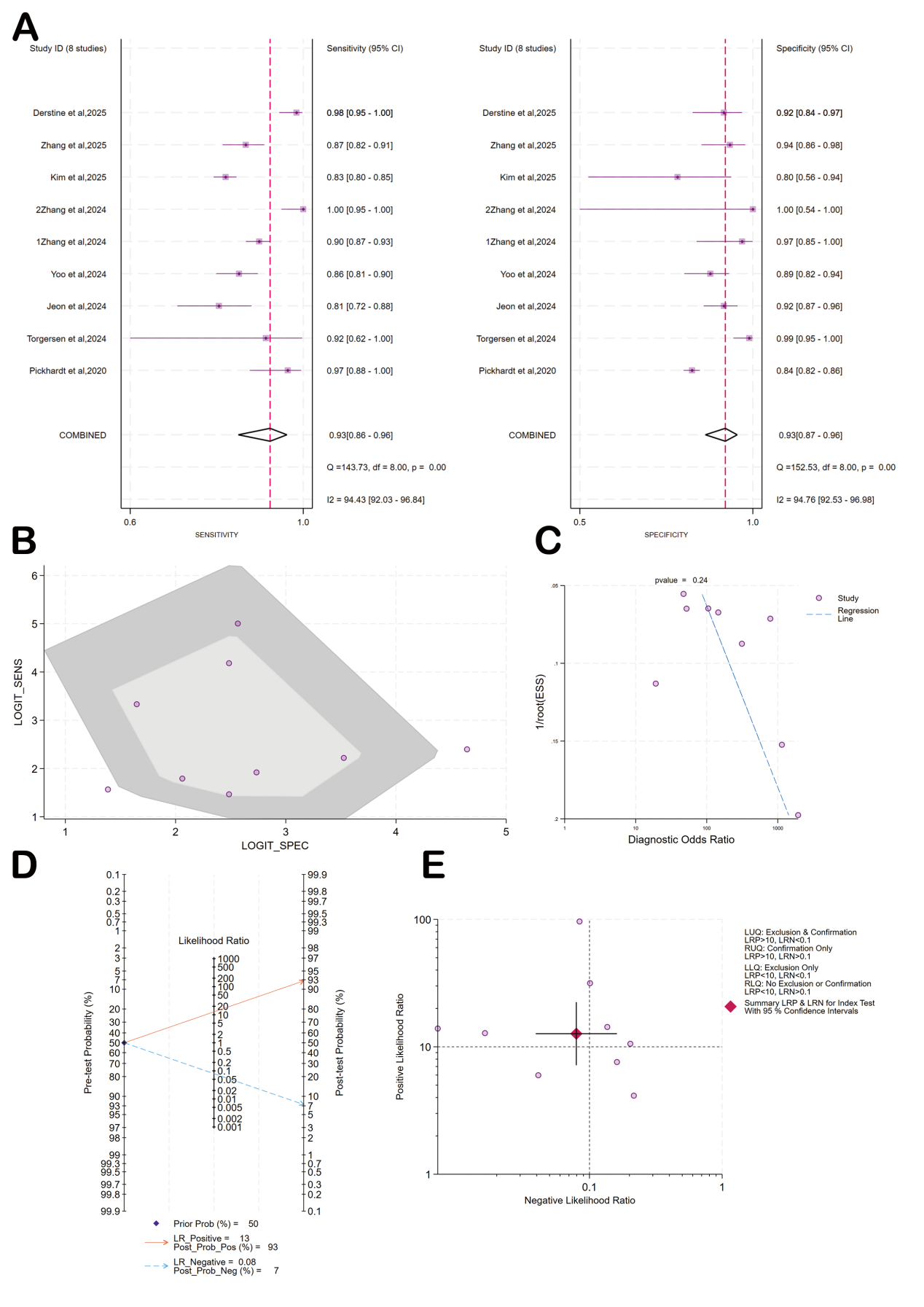
Supplementary Figure S7. Diagnostic performance summary for studies based on computed tomography (CT) imaging: forest plots, bivariate boxplot, Deeks' funnel plot, Fagan's nomogram, and clinical application plot.**

A) Forest plots of sensitivity and specificity for CT imaging (8 studies, 9 datasets). B) Bivariate boxplot illustrating distribution and heterogeneity. C) Deeks’ funnel plot assessing potential publication bias. D) Fagan’s nomogram depicting post-test probability. E) Clinical application plot of LRP and LRN.

**
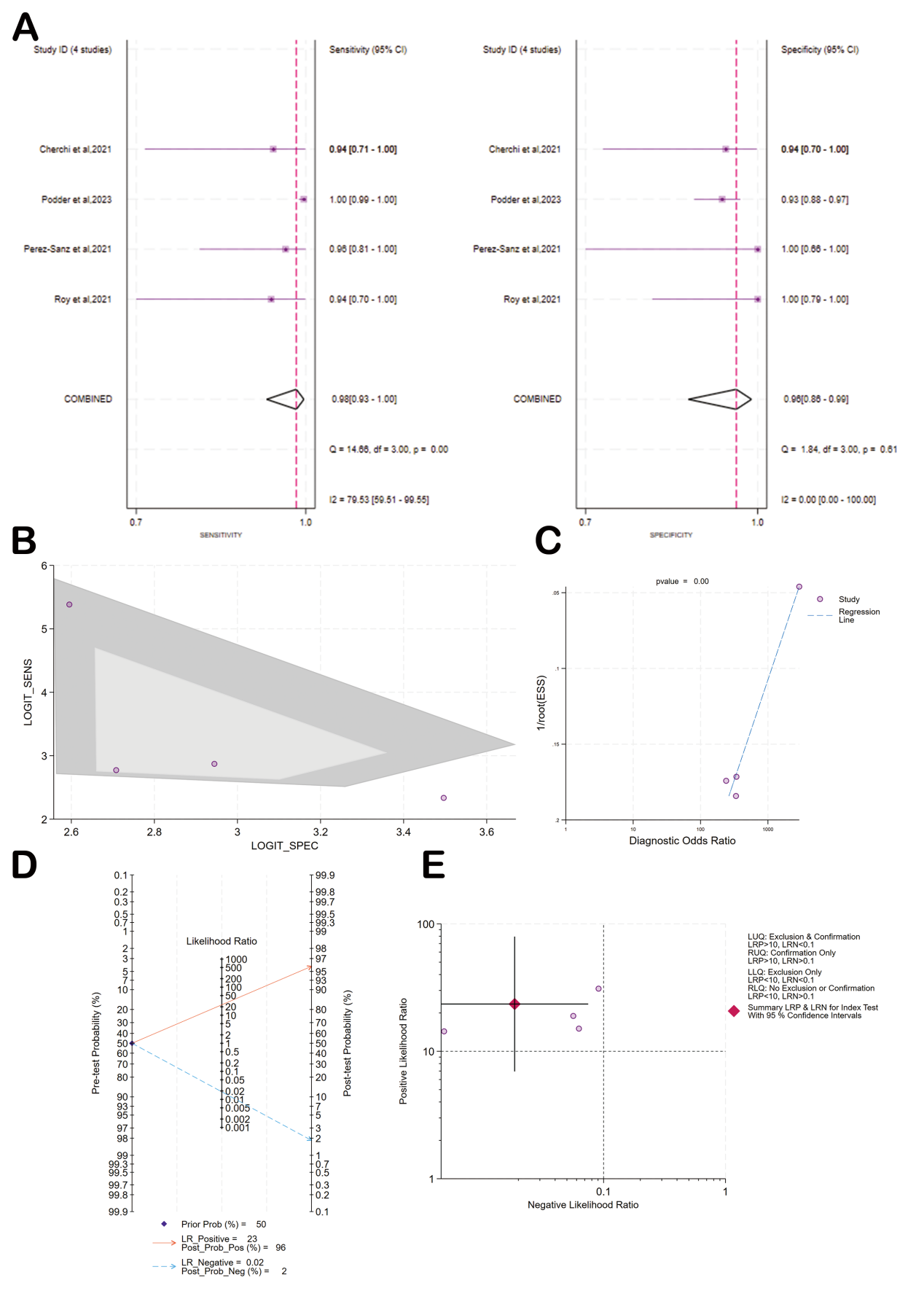
Supplementary Figure S8. Diagnostic performance summary for studies based on pathological imaging: forest plots, bivariate boxplot, Deeks' funnel plot, Fagan's nomogram, and clinical application plot.**

A) Forest plots of sensitivity and specificity for pathology imaging (4 studies, 4 datasets). B) Bivariate boxplot illustrating distribution and heterogeneity. C) Deeks’ funnel plot assessing potential publication bias. D) Fagan’s nomogram depicting post-test probability. E) Clinical application plot of LRP and LRN.

**
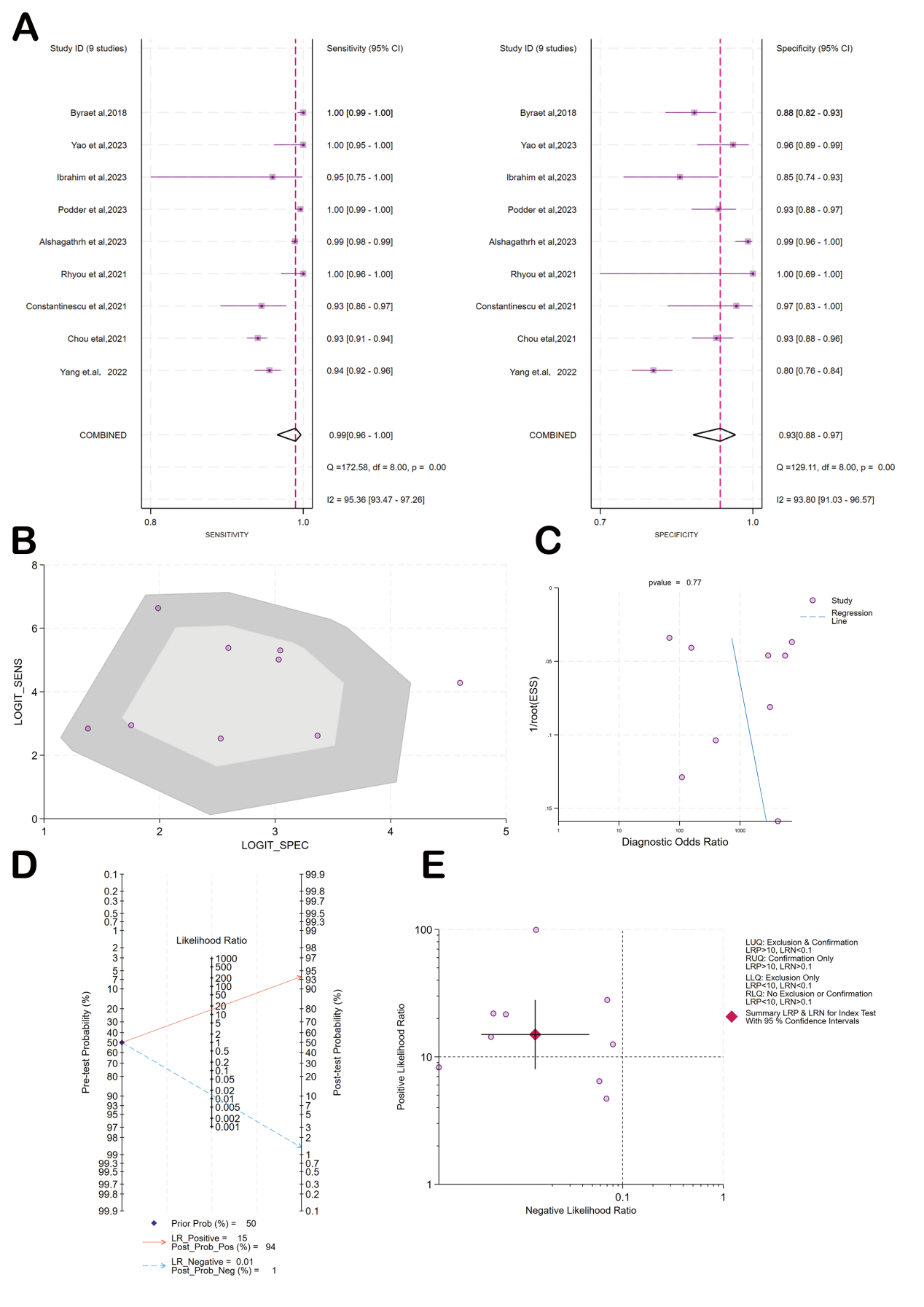
Supplementary Figure S9. Diagnostic performance summary for studies employing transfer learning (TL): forest plots, bivariate boxplot, Deeks' funnel plot, Fagan's nomogram, and clinical application plot.**

A) Forest plots of sensitivity and specificity for studies employing TL (9 studies, 9 datasets). B) Bivariate boxplot illustrating distribution and heterogeneity. C) Deeks’ funnel plot assessing potential publication bias. D) Fagan’s nomogram depicting post-test probability. E) Clinical application plot of LRP and LRN.

**
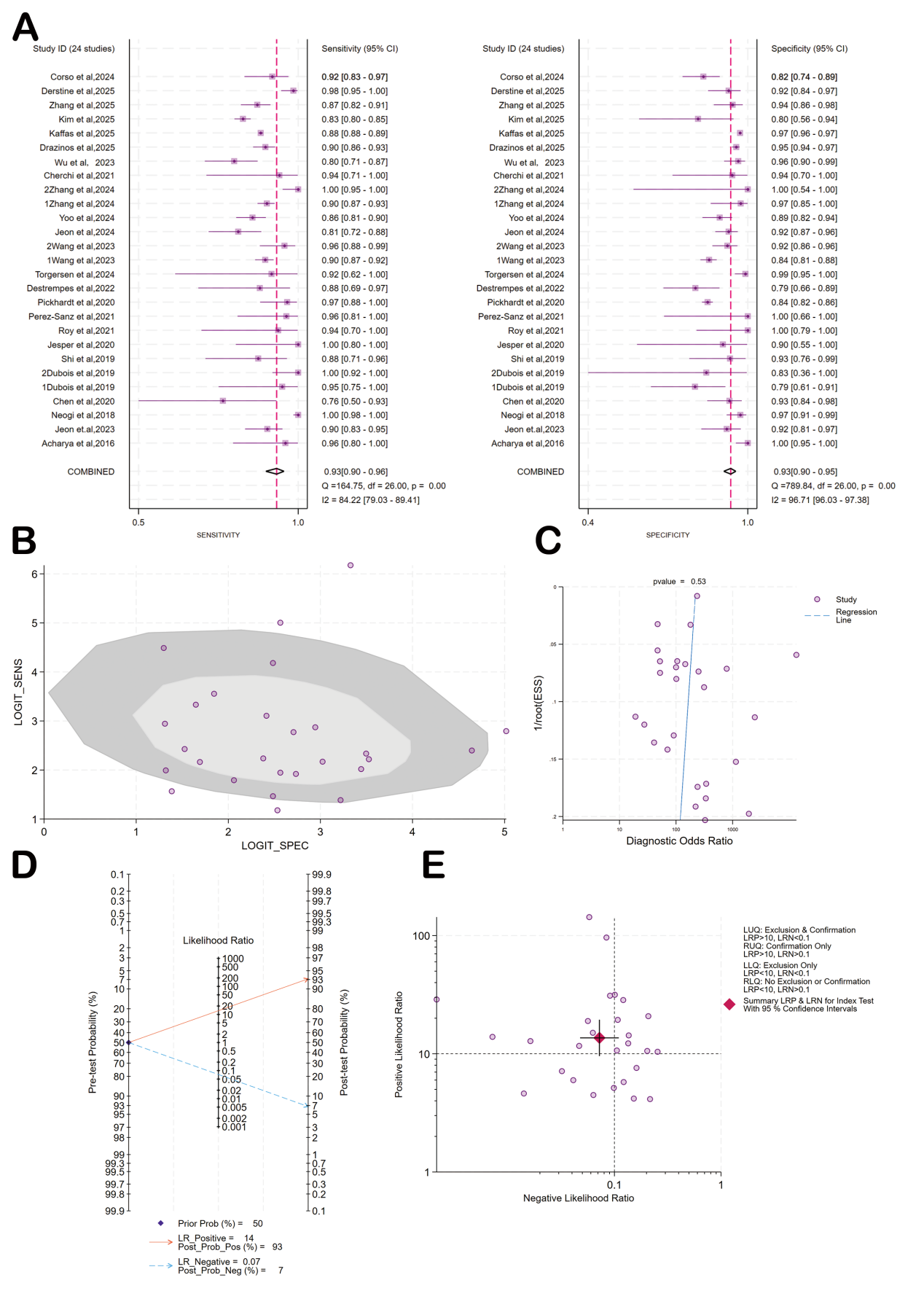
Supplementary Figure S10. Diagnostic performance summary for studies not employing transfer learning (TL): forest plots, bivariate boxplot, Deeks' funnel plot, Fagan's nomogram, and clinical application plot.**

A) Forest plots of sensitivity and specificity for studies not employing TL (24 studies, 26 datasets). B) Bivariate boxplot illustrating distribution and heterogeneity. C) Deeks’ funnel plot assessing potential publication bias. D) Fagan’s nomogram depicting post-test probability. E) Clinical application plot of LRP and LRN.

**
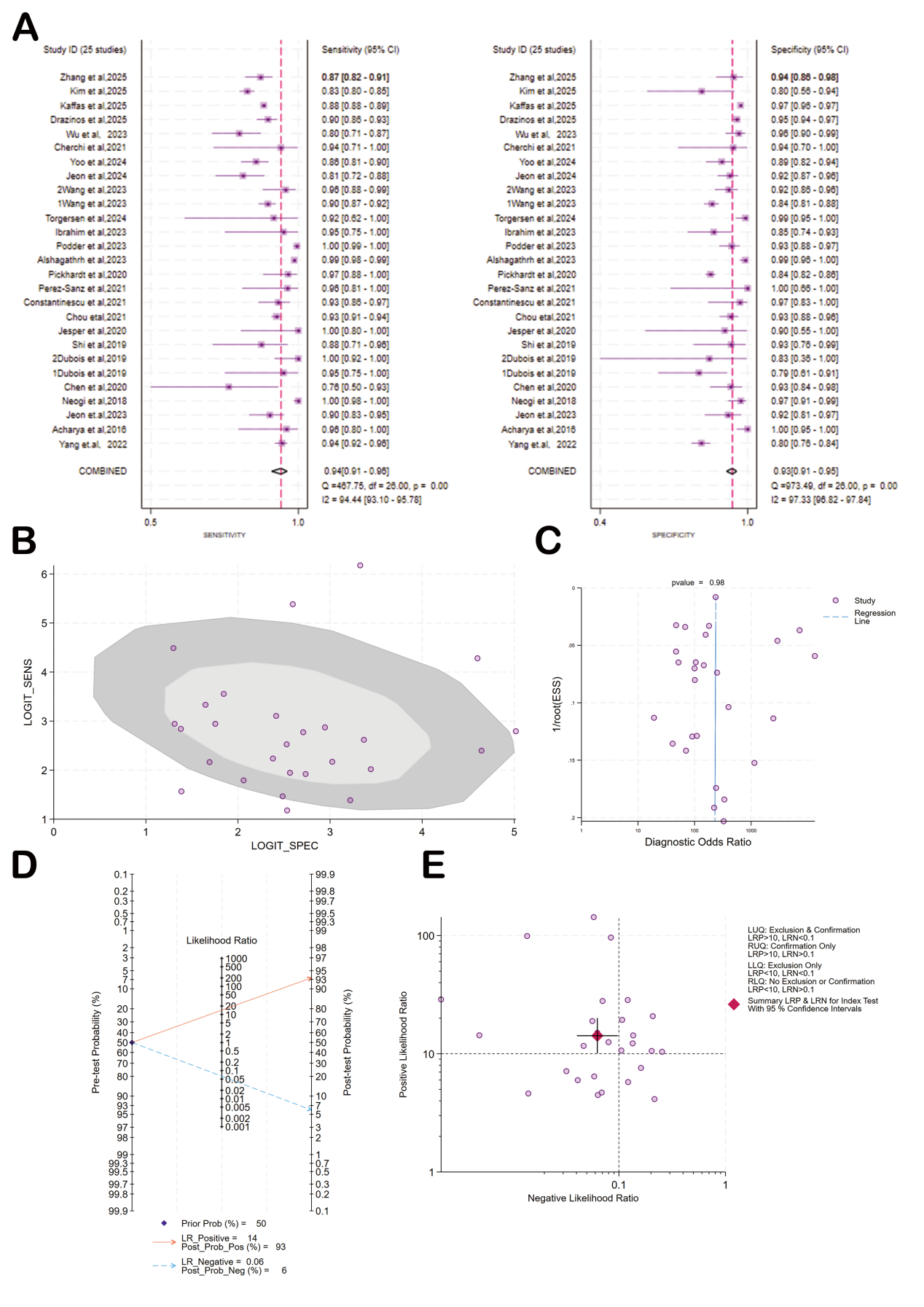
Supplementary Figure S11. Diagnostic performance summary for single-center studies: forest plots, bivariate boxplot, Deeks' funnel plot, Fagan's nomogram, and clinical application plot.**

A) Forest plots of sensitivity and specificity for single-center studies (25 studies, 26 datasets). B) Bivariate boxplot illustrating distribution and heterogeneity. C) Deeks’ funnel plot assessing potential publication bias. D) Fagan’s nomogram depicting post-test probability. E) Clinical application plot of LRP and LRN.

**
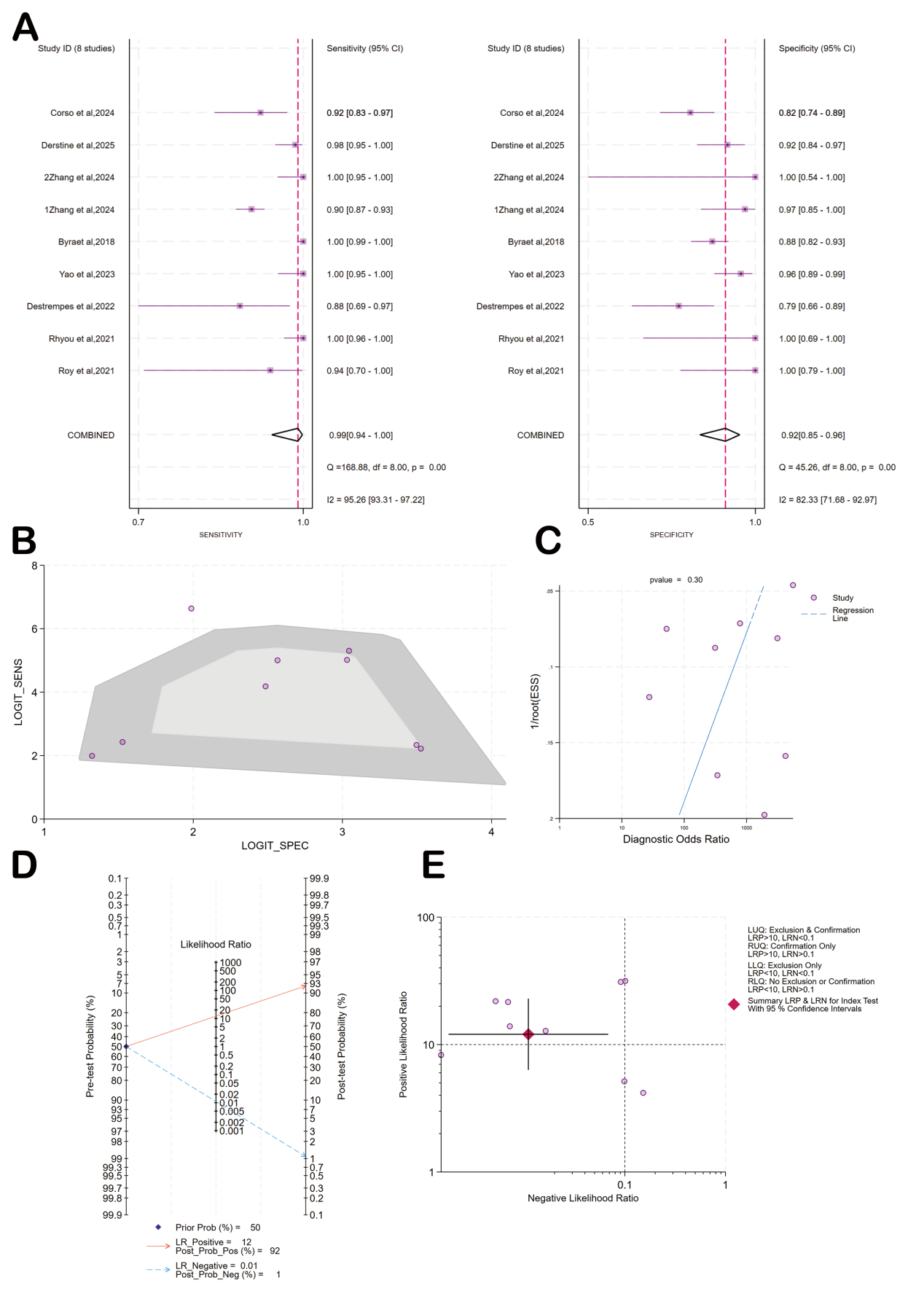
Supplementary Figure S12. Diagnostic performance summary for multi-center studies: forest plots, bivariate boxplot, Deeks' funnel plot, Fagan's nomogram, and clinical application plot.**

A) Forest plots of sensitivity and specificity for multi-center studies (8 studies, 9 datasets). B) Bivariate boxplot illustrating distribution and heterogeneity. C) Deeks’ funnel plot assessing potential publication bias. D) Fagan’s nomogram depicting post-test probability. E) Clinical application plot of LRP and LRN.

**
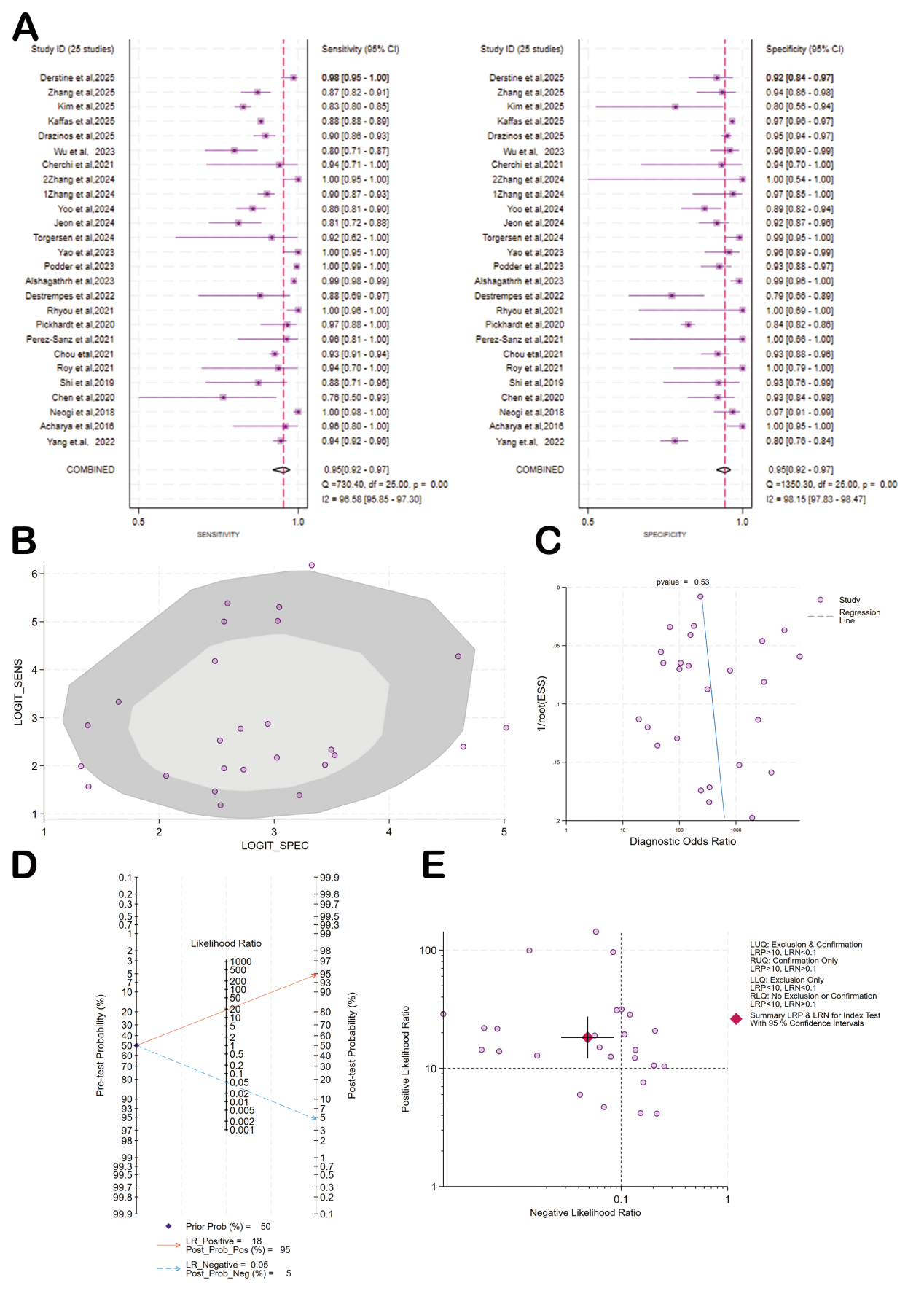
Supplementary Figure S13. Diagnostic performance summary for retrospective studies: forest plots, bivariate boxplot, Deeks' funnel plot, Fagan's nomogram, and clinical application plot.**

A) Forest plots of sensitivity and specificity for retrospective studies (25 studies, 26 datasets). B) Bivariate boxplot illustrating distribution and heterogeneity. C) Deeks’ funnel plot assessing potential publication bias. D) Fagan’s nomogram depicting post-test probability. E) Clinical application plot of LRP and LRN.

**
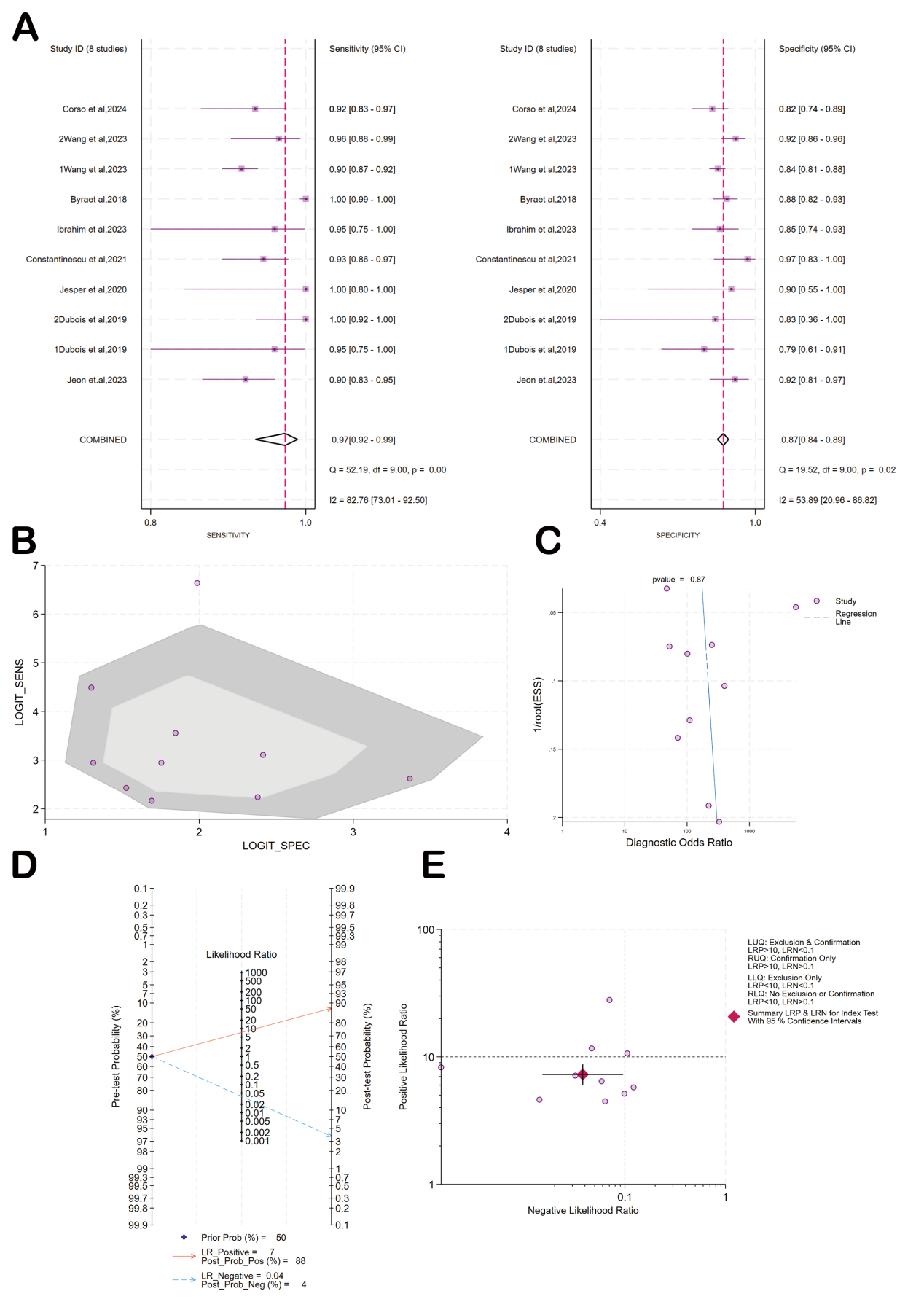
Supplementary Figure S14. Diagnostic performance summary for prospective studies: forest plots, bivariate boxplot, Deeks' funnel plot, Fagan's nomogram, and clinical application plot.**

A) Forest plots of sensitivity and specificity for prospective studies (8 studies, 9 datasets). B) Bivariate boxplot illustrating distribution and heterogeneity. C) Deeks’ funnel plot assessing potential publication bias. D) Fagan’s nomogram depicting post-test probability. E) Clinical application plot of LRP and LRN.

**
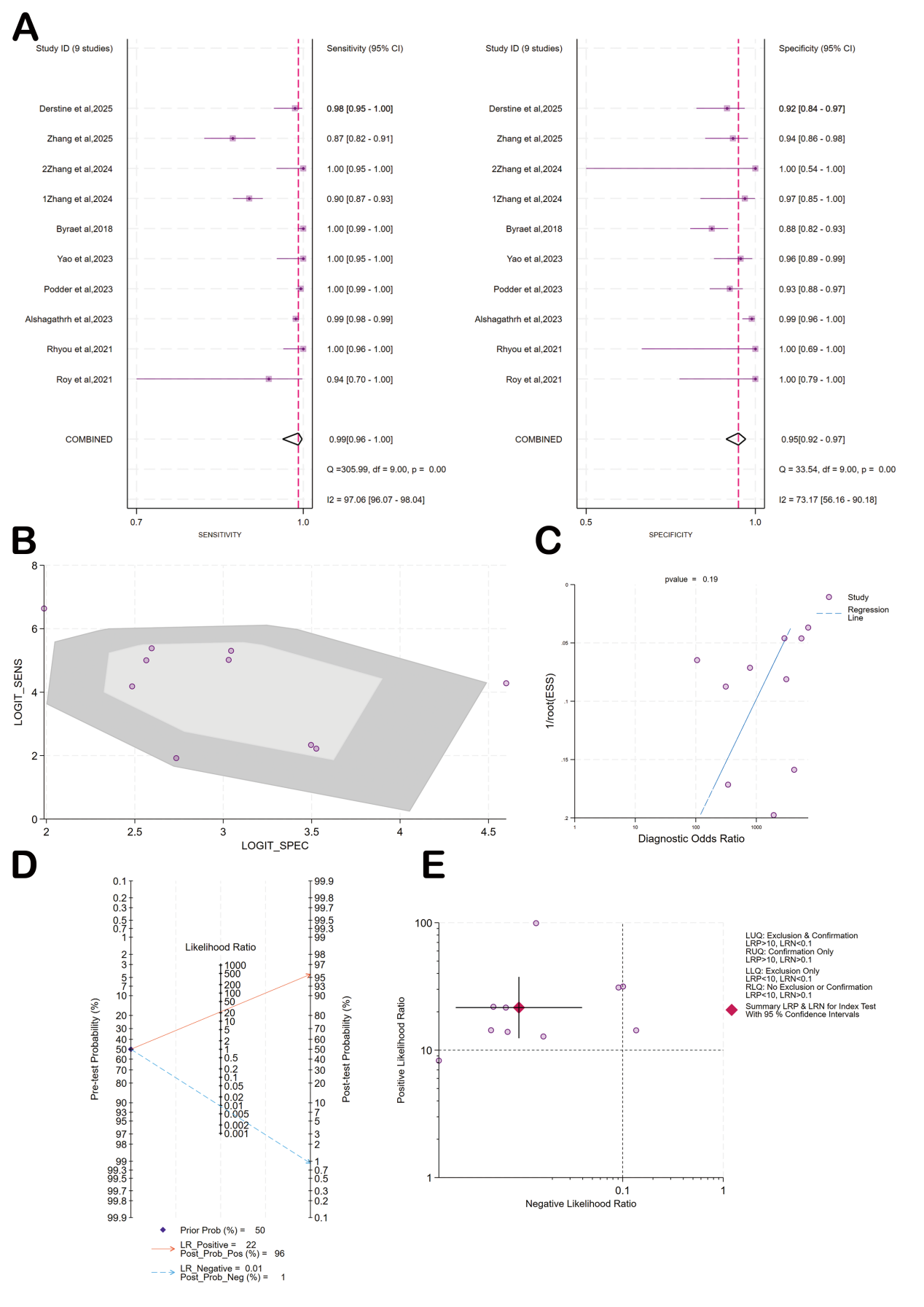
Supplementary Figure S15. Diagnostic performance summary for studies with publicly available data: forest plots, bivariate boxplot, Deeks' funnel plot, Fagan's nomogram, and clinical application plot.**

A) Forest plots of sensitivity and specificity for studies with available data (9 studies, 10 datasets). B) Bivariate boxplot illustrating distribution and heterogeneity. C) Deeks’ funnel plot assessing potential publication bias. D) Fagan’s nomogram depicting post-test probability. E) Clinical application plot of LRP and LRN.

**
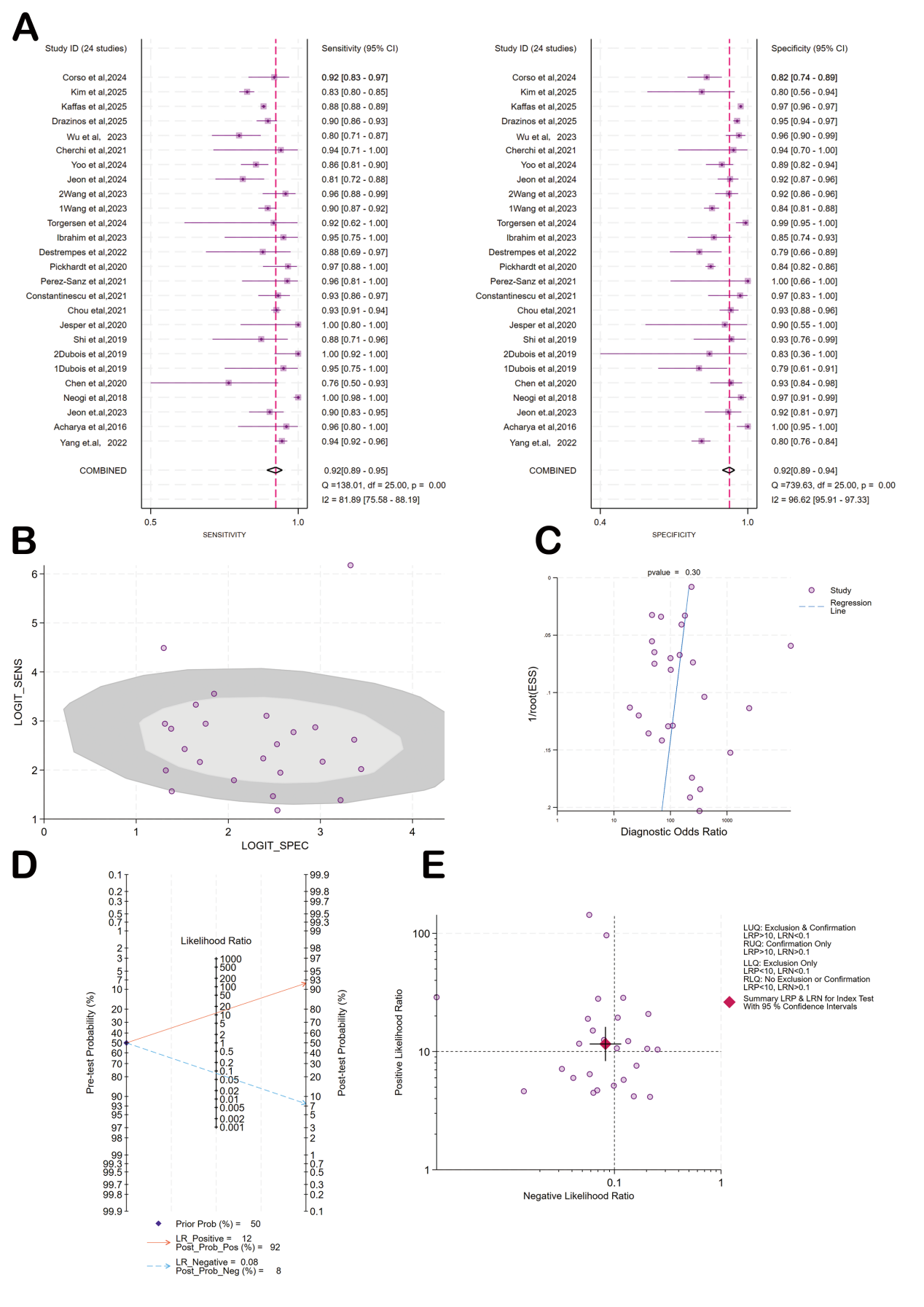
Supplementary Figure S16. Diagnostic performance summary for studies without publicly available data: forest plots, bivariate boxplot, Deeks' funnel plot, Fagan's nomogram, and clinical application plot.**

A) Forest plots of sensitivity and specificity for studies with unavailable data (24 studies, 25 datasets). B) Bivariate boxplot illustrating distribution and heterogeneity. C) Deeks’ funnel plot assessing potential publication bias. D) Fagan’s nomogram depicting post-test probability. E) Clinical application plot of LRP and LRN.
